# *Lycium barbarum* polysaccharide reverses drug resistance in oxaliplatin-resistant colon cancer cells by inhibiting PI3K/AKT-dependent phosphomannose isomerase

**DOI:** 10.3389/fphar.2024.1367747

**Published:** 2024-03-21

**Authors:** Lijun Ma, Fangfang Ai, Hongyan Xiao, Fang Wang, Lei Shi, Xuehong Bai, Yongzhao Zhu, Wenping Ma

**Affiliations:** ^1^ Department of Human Anatomy, Histology and Embryology, School of Basic Medicine, Ningxia Medical University, Yinchuan, China; ^2^ Key Laboratory of Ningxia Ethnomedicine Modernization of Ministry of Education, Ningxia Medical University, Yinchuan, China; ^3^ School of Clinical Medicine, Ningxia Medical University, Yinchuan, China; ^4^ People’s Hospital of Ningxia Hui Autonomous Region, Yinchuan, China; ^5^ School of Biological Science and Engineering, North Minzu University, Yinchuan, China

**Keywords:** PMI, ABCG2, *Lycium barbarum* polysaccharide, colon cancer, drug resistance

## Abstract

**Objective:** Here, we aimed to explore the effect of LBP in combination with Oxaliplatin (OXA) on reversing drug resistance in colon cancer cells through *in vitro* and *in vivo* experiments. We also aimed to explore the possible mechanism underlying this effect. Finally, we aimed to determine potential targets of *Lycium barbarum* polysaccharide (LBP) in colon cancer (CC) through network pharmacology and molecular docking.

**Methods:** The invasion ability of colon cancer cells was assessed using the invasion assay. The migration ability of these cells was assessed using the migration assay and wound healing assay. Cell cycle analysis was carried out using flow cytometry. The expression levels of phosphomannose isomerase (PMI) and ATP-binding cassette transport protein of G2 (ABCG2) proteins were determined using immunofluorescence and western blotting. The expression levels of phosphatidylinositol3-kinase (PI3K), protein kinase B (AKT), B-cell lymphoma 2 (Bcl-2), and BCL2-Associated X (Bax) were determined using western blotting. Forty BALB/c nude mice purchased from Weitong Lihua, Beijing, for the *in vivo* analyses. The mice were randomly divided into eight groups. They were administered HCT116 and HCT116-OXR cells to prepare colon cancer xenograft models and then treated with PBS, LBP (50 mg/kg), OXA (10 mg/kg), or LBP + OXA (50 mg/kg + 10 mg/kg). The tumor weight and volume of treated model mice were measured, and organ toxicity was evaluated using hematoxylin and eosin staining. The expression levels of PMI, ABCG2, PI3K, and AKT proteins were then assessed using immunohistochemistry. Moreover, PMI and ABCG2 expression levels were analyzed using immunofluorescence and western blotting. The active components and possible targets of LBP in colon cancer were explored using *in silico* analysis. GeneCards was used to identify CC targets, and an online Venn analysis tool was used to determine intersection targets between these and LBP active components. The PPI network for intersection target protein interactions and the PPI network for interactions between the intersection target proteins and PMI was built using STRING and Cytoscape. To obtain putative targets of LBP in CC, we performed GO function enrichment and KEGG pathway enrichment analyses.

**Results:** Compared with the HCT116-OXR blank treatment group, both invasion and migration abilities of HCT116-OXR cells were inhibited in the LBP + OXA (2.5 mg/mL LBP, 10 μΜ OXA) group (*p <* 0.05). Cells in the LBP + OXA (2.5 mg/mL LBP, 10 μΜ OXA) group were found to arrest in the G1 phase of the cell cycle. Knockdown of PMI was found to downregulate PI3K, AKT, and Bcl-2 (*p <* 0.05), while it was found to upregulate Bax (*p <* 0.05). After treatment with *L. barbarum* polysaccharide, 40 colon cancer subcutaneous tumor models showed a decrease in tumor size. There was no difference in the liver index after LBP treatment (*p* > 0.05). However, the spleen index decreased in the OXA and LBP + OXA groups (*p* < 0.05), possibly as a side effect of oxaliplatin. Immunohistochemistry, immunofluorescence, and western blotting showed that LBP + OXA treatment decreased PMI and ABCG2 expression levels (*p* < 0.05). Moreover, immunohistochemistry showed that LBP + OXA treatment decreased the expression levels of PI3K and AKT (*p* < 0.05). Network pharmacology analysis revealed 45 active LBP components, including carotenoids, phenylpropanoids, quercetin, xanthophylls, and other polyphenols. It also revealed 146 therapeutic targets of LBP, including AKT, SRC, EGFR, HRAS, STAT3, and MAPK3. KEGG pathway enrichment analysis showed that the LBP target proteins were enriched in pathways, including cancer-related signaling pathways, PI3K/AKT signaling pathway, and IL-17 signaling pathways. Finally, molecular docking experiments revealed that the active LBP components bind well with ABCG2 and PMI.

**conclusion:** Our *in vitro* experiments showed that PMI knockdown downregulated PI3K, AKT, and Bcl-2 and upregulated Bax. This finding confirms that PMI plays a role in drug resistance by regulating the PI3K/AKT pathway and lays a foundation to study the mechanism underlying the reversal of colon cancer cell drug resistance by the combination of LBP and OXA. Our *in vivo* experiments showed that LBP combined with oxaliplatin could inhibit tumor growth. LBP showed no hepatic or splenic toxicity. LBP combined with oxaliplatin could downregulate the expression levels of PMI, ABCG2, PI3K, and AKT; it may thus have positive significance for the treatment of advanced metastatic colon cancer. Our network pharmacology analysis revealed the core targets of LBP in the treatment of CC as well as the pathways they are enriched in. It further verified the results of our *in vitro* and *in vivo* experiments, showing the involvement of multi-component, multi-target, and multi-pathway synergism in the drug-reversing effect of LBP in CC. Overall, the findings of the present study provide new avenues for the future clinical treatment of CC.

## Graphical Abstract

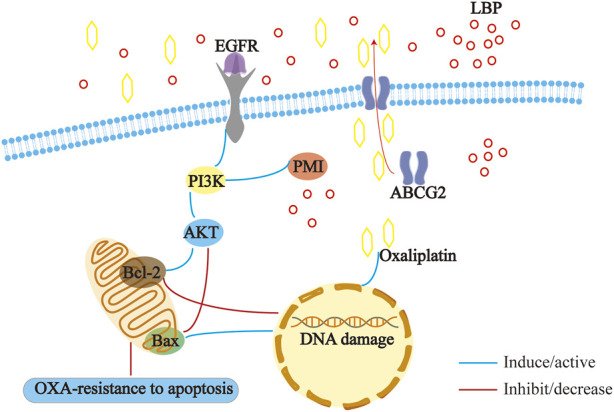

## Introduction

According to GLOBOCAN 2023 findings, with the global cancer burden continuously increasing, cancer has become the second leading cause of death, ranking second only to heart disease, and colorectal cancer ranks third in terms of both cancer incidence and mortality in both men and women (Siegel et al., 2023). Among the available clinical chemotherapy regimens for colon cancer, oxaliplatin-based and irinotecan-based chemotherapy regimens remain the first-line chemotherapies (Yamazaki et al., 2016; Guan et al., 2020). However, drug resistance, metastasis, and recurrence are prominent challenges in the clinical treatment of colorectal cancer. Therefore, it is important to find a solution for oxaliplatin resistance in colon cancer.

In the present study, we focused on elucidating the mechanism of action of LBP in the treatment of colon cancer through network pharmacology. We investigated the effect of treatment with LBP on the migration and invasion abilities of colon cancer cells. Cell cycle analysis was performed using flow cytometry. To explore the mechanism underlying the therapeutic effect of LBP in colon cancer, we detected PI3K, AKT, Bax, and Bcl-2 expression levels after knocking down PMI. In *in vivo* experiments, the tumor size was recorded after tumor-bearing mice were treated with LBP combined with oxaliplatin. We also examined the toxic effects of LBP treatment on the visceral heart, liver, spleen, lungs, and kidneys as well as the expression levels of PMI, ABCG2, PI3K, and AKT in mouse tumor tissues. By combining the results of network pharmacology with those of *in vivo* and *in vitro* studies, we aimed to provide here, new avenues for the treatment of drug-resistant colorectal cancer.

*Lycium barbarum* polysaccharide is an important functional component of the red fruit of *L. barbarum*, which has a variety of benefits, including lowering blood glucose and lipid levels, immunomodulation, anti-cancer effects, anti-aging effects, anti-fatigue effects, and anti-glaucoma effects (Jiao et al., 2016; Zhang Y. et al., 2020). A large number of studies have also demonstrated the anti-tumor activity of *L. barbarum* polysaccharide (Miao et al., 2010; Zhang et al., 2013; Georgiev et al., 2019; Ran et al., 2020; Yang et al., 2022). However, the specific mechanism underlying this activity is still unclear. Therefore, the present study was conducted to investigate the anti-tumor effects of *L. barbarum* polysaccharide *in vitro* and *in vivo*.

Phosphomannose isomerase (PMI) is a zinc finger-dependent enzyme that catalyzes the reversible isomerization of mannose 6-phosphate (M6P) and fructose 6-phosphate (F6P). M6P is required for the mannose glycosylation of various microbial cell wall proteins. The deletion or mutation of the PMI gene can thus lead to defective microbial cell wall synthesis and eventually death; therefore, *PMI* can be used as an antibacterial drug target. PMI is also involved in energy metabolism, as its catalysis product F6P can be oxidized by glycolysis. In eukaryotes, PMI deletion leads to the accumulation of F6P or M6P, defective glycosylation, and induction of P53 expression (Bangera et al., 2019). Rapid cell proliferation in early development and cancer relies on glucose metabolism to promote macromolecular biosynthesis. Metabolic enzymes are thought to be regulators of this glycolysis-driven macromolecular biosynthesis, and this process is known as the Warburg effect. Shtraizent, N provided mechanistic evidence that PMI deletion induces p53 expression, identifying PMI as a novel regulator of p53 and Warburg metabolism (Shtraizent et al., 2017).

Multiple drug resistance-related proteins and cancer stem cells (CSCs) play an important role in the metastasis and recurrence of colon cancer. ABCG2 is a 72-kDa “half transporter.” Physiologically, ABC transporter proteins are expressed in the small intestine, liver, kidney, blood-brain barrier, choroid plexus, testes, placenta, and other important biological barriers (Hu et al., 2016). ABCG2 is the second member of the ABC (ATP transporter cassette) transporter G family and is also known as breast cancer resistance protein (BCRP) (Huang et al., 2013). In addition to ABCG2, two other members of the ABC transporter family, P-gp and MDR-associated proteins, account for the majority of human multidrug resistance (An and Ongkeko, 2009).

ABCG2 also plays an important role in the stem cell field (Mao and Unadkat, 2015). Haraguchi et al., 2006 revealed that ABCG2-expressing gastrointestinal cancer cells exhibit stem cell properties, defining this group of cells as side population cells. Conventional chemotherapy induces apoptosis by breaking DNA or inhibiting mitosis, and, since this treatment is only effective against highly proliferating cancer cells, slowly proliferating CSCs evade the chemotherapeutic drug, leading to recurrence (Mohan et al., 2021). High expression levels of the efflux protein ABCG2 and the apoptosis-associated Bcl-2 protein family represent drug resistance mechanisms in colon cancer cells based on transport and a lack of transport, respectively (Hu et al., 2016). ABCG2, an important marker for colon cancer stem cells, is highly expressed in drug-resistant cells. Therefore, the present study investigated whether LBP could affect the expression levels of ABCG2 as well as the Bcl-2 protein family and explore possible mechanisms underlying these effects in order to provide a theoretical basis for clinical treatment of colon cancer.

## Materials and methods

### Materials

HCT116 and HCT116-OXR cell lines were purchased from ATCC. 40 BALB/c nude mice (average weights of 14.27 ± 1.25 g) were purchased from Beijing Wei tong Lihua. Matrigel was purchased from Shanghai Yuchun Biotechnology (B-P-00002, Shanghai, China). The hematoxylin and eosin staining kit, 4% poly formaldehyde, and DAB kit were purchased from Zhongshan Jinqiao (BSBA-4025 and PV-9000, Beijing, China). McCoy’s 5A and DMEM cell culture media were purchased from Procell Biotechnology (PM150710 and PM150210, Wuhan, China). Fetal bovine serum was purchased from GIBCO (10099141C, Carlsbad, CA, United States). Oxaliplatin was purchased from MCE (HY-17371, Shanghai, China). Lycium polysaccharide was purchased from Ningxia Tianren Lycium Biotechnology (20170501, Ningxia, China). The mouse monoclonal antibodies, MPI and ABCG2, were purchased from Santa Cruz Biotechnology (sc-393484 and sc-377176, Dallas, TX, United States). Rabbit polyclonal antibodies PI3K and AKT were purchased from Cell Signaling Technology (4249, 4691, Danvers, MA, United States). The rabbit polyclonal antibodies, Bcl-2 and β-tubulin and the horseradish peroxidase-labeled goat anti-mouse and goat anti-rabbit IgG antibodies were purchased from Affinity Biosciences. The transwell chambers and matrigel were purchased from Corning (3422 and 354248, Corning, NY, United States). The 0.1% crystal violet, 4% poly formaldehyde, and anti-fluorescence quench sealed tablets containing DAPI were purchased from Solarbio (G1063, P1110 and S2110-5, Beijing, China). Goat anti-mouse IgG labeled with rhodamine were purchased from Zhongshan Jinqiao, Beijing (ZF-0313, Beijing, China). The BCA kit, ECL detection kit, whole protein extraction kit, and cell cycle analysis kit were purchased from Jiangsu Key GEN Biology (KGP902, KGP1121, KGP250, and KGA512, Jiangsu, China). Lipo3000 liposome transfection reagent was purchased from Thermo Fisher Scientific (L3000015, Waltham, MA, United States). The reverse transcription kit and SYBR fluorescent quantitative kit were purchased from TIANGEN (KR118-02 and FP205-02, Beijing, China).

### Methods

#### Cell culture

HCT116 and HCT116-OXR human colon cancer cells were cultured in a sterile incubator at 37°C with 5% CO_2_, and the culture medium was changed every 3 days. HCT116 cells were cultured in McCoy’s 5A medium containing 10% fetal bovine serum, and HCT116-OXR cells were cultured in DMEM containing 10% fetal bovine serum. The treatment groups for the *in vitro* studies were as follows: HCT116 control group, HCT116-OXR blank treatment group, LBP treatment group (2.5 mg/mL), oxaliplatin treatment group (20 μM), LBP (2.5 mg/mL) combined with oxaliplatin (20 μM) treatment group.

## Design for animal experiments

All mice were raised in the animal experiment center of Ningxia Medical University with permit No. IACUC-NYLAC-181. The mice were randomly divided into eight groups. Then, xenografted colon cancer mouse models were created by injecting mice with HCT116 cells or HCT116-OXR cells (four groups each) through subcutaneous injection. The mice were then treated with PBS, LBP (50 mg/kg), OXA (10 mg/kg), or LBP + OXA (50 mg/kg + 10 mg/kg). LBP was administered by gavage once every 2 days for 14 days. OXA was administered through intraperitoneal injection once every 2 days for 14 days.

### Xenografted colon cancer mouse models

Matrigel C (10×) was diluted to matrigel C (0.5×) with cell culture medium containing 20% serum. Matrigel A (2×) was diluted to matrigel A (1×) with cell culture medium containing 20% serum. Then, Matrigel C was heated in a 37°C water bath for 10 min to prevent coagulation. Next, the cells were digested by pancreatic enzymes and centrifuged at 1,000 rpm for 5 min. The cell precipitate was then collected and mixed with C buffer solution (0.5×) to obtain a cell concentration of 6 × 10^7^/mL. Solution A was then added to solution C mixture of cells before injection at a ratio of 2:1. The final concentration of the cell suspension was 2 × 10^7^/mL. Each mouse was injected with 100 μL of cell suspension.

The pentobarbital (50 mg/kg) dose was calculated according to the body weight of the mice. After the animals were anesthetized, the cancer cells were injected into their right axilla. The mice were randomly divided into eight groups. Four groups were injected with HCT116 cells and four were injected with HCT116-OXR cells. Hundred microliters of the cell suspension were injected using a 1-mL syringe at room temperature. After successful injection, we waited for the mice to wake up successfully. Then, tumor formation was observed for 1–3 weeks. Tumor models with tumor volumes of about 100 mm^3^ were used for further study. Tumor size was measured with vernier calipers every 2 days. Tumor volume was calculated as 1/2 ab^2^. Mouse weight was also recorded. Tumor weight was measured at the time of sampling. Organ coefficients were calculated as follows: weight of organ (g)/body weight (g) × 100%.

### Migration assay

Transwell chambers were placed in a 24-well plate; 200 μL of the serum-free medium was added to the upper chamber, 800 μL of serum-free medium was added to the lower chamber, and the plate was incubated in a cell incubator for 2 h. The drug-treated cells were then digested and transferred to EP tubes and centrifuged at 1000 *g* for 5 min. The supernatant was aspirated and discarded, and cell counting was performed after the cells were resuspended in serum-free medium. Medium containing 10% FBS was added to another row of the 24-well plate. A total of 2.5 × 10^4^–5 × 10^4^ cells were seeded in the upper transwell chamber, and the chamber was placed in a well containing medium with 10% FBS. The 24-well plates were then placed in an incubator after all treatments were completed; at the end of the incubation period, the culture medium was discarded, and the chamber was washed thrice with precooled PBS. The cells attached to the floor of the upper chamber were fixed at room temperature for 20 min after precooled paraformaldehyde was added to both its inner and outer surfaces. After fixation, the PFA was aspirated and discarded, and the chamber was washed thrice with precooled PBS. the chamber was then stained in the dark for 20 min with 0.1% crystal violet solution. After staining, the chamber was washed five times with precooled PBS, until the PET film of the chamber was washed clean. A clean cotton swab was then used to gently wipe the chamber to remove excess cells. The chamber was then inverted onto a clean bench and blow dried. Photographs of the fixed cells were then obtained using an inverted microscope in 3–5 fields randomly selected fields.

### Invasion assay

Fresh matrigel was diluted with precooled serum-free matrigel at a ratio of 1:9. Hundred microliters of this matrigel solution was added to each transwell chamber, and the chambers were then placed in 24-well plates and incubated overnight in a CO_2_ incubator at 37°C. The excess coating solution was aspirated with a pipetting gun after the incubation was complete. Fifty microliters of serum-free medium were then added to each well, and the plate was placed in an incubator at 37°C for 30 min. Next, the starved cells that had been cultured in serum-free medium for 12–24 h were digested, and about 7.5 × 10^4^ cells were counted; 200 μL of the cell suspension was seeded in a transwell chamber after repeated pipetting to ensure homogeneity. Eight hundred microliters of antibiotic-free medium containing 10% FBS were added to the lower transwell chamber. After routine culture, the cells were fixed with 4% paraformaldehyde, stained with crystal violet for 15–30 min, counted under a microscope, and photographed under a fluorescence microscope.

### Scratch test

Cells were seeded in new 6-well plates (30 × 10^5^ cells/well) for 24 h and cultured to 80%–90% confluence. Subsequently, a straight scratch was created using a 200-μL sterile pipette tip. Cells were then cultured in serum-free medium in a humidified incubator containing 5% CO_2_ at 37°C. Finally, the migration distance was observed, and images were captured after 0 h and 48 h. Scratch areas were recorded using Auto2 (Thermo Fisher) and analyzed using ImageJ. The width of the cell scratch was measured, and the wound healing rate was calculated as follows: wound healing rate (%) = [(scratch distance of the treatment group—0-h scratch width)/0-h scratch width] × 100%

### Hematoxylin and eosin staining

Heart, liver, spleen, lung, and kidney tissues were fixed in 4% formaldehyde solution for 48 h, embedded in paraffin, and cut into 4-μm sections. This was followed by standard H&E procedures. The slides were observed under a microscope to determine the cell morphology and histopathological changes.

### Immunohistochemistry

After the tumor tissues were fixed for 48 h, they were paraffin-embedded. Then, they were cut into 4-μm sections. Antigen retrieval was then performed using citrate buffer (pH 6.0) after paraffin was removed from the slides. Next, the slides were incubated with endogenous peroxidase to quench endogenous peroxidase activity. Then, the slides were incubated with the primary antibody (PMI 1:200 or ABCG2 1:200) overnight at 4°C. The following day, the slides were incubated with HRP-conjugated anti-goat secondary antibody (1:200), followed by DAB staining. Hematoxylin was used for repeat staining, and dehydration and sealing were routinely performed. The target protein-positive area was analyzed using ImageJ.

### Immunofluorescence staining

In case of cell-containing slides, the procedure employed was as follows. On the first day, the appropriate number of cells in each group was inoculated on appropriately-sized sterile glass slides in a sterile 24-well plate, The next day, the cells were found adhered to the walls of the wells and grew on the slides in good condition and at a moderate density. After different treatments, the medium in each well was aspirated and discarded, and the slides were washed gently with PBS thrice. An appropriate amount of 4% paraformaldehyde was then added into each well to fix the cells for 20 min. Then, the slides were washed gently thrice with PBS. An appropriate amount of PBS containing 3% goat serum was added to block for 1 h at room temperature. Primary antibodies were then added to the slides, and the slides were incubated at 4°C overnight. The dilution ratios of the antibodies were as follows: mouse anti-human PMI (1:100) and mouse anti-human ABCG2 (1:100). Next, the slides were washed gently with PBS thrice. Fluorescent secondary antibodies were added to the slides, which were then incubated at room temperature for 1 h in the dark. The dilution ratio of the rhodamine-labeled goat anti-mouse secondary antibody was 1:200. Next, the slides were washed gently with PBS thrice. After DAPI staining for 3 min, the slides were sealed with neutral resin and observed under a fluorescence microscope.

In case of tumor tissue section-containing slides, the procedure employed was as follows. The tumor tissue slides were placed in an oven at 60°C for 2 h. Then, the paraffin was removed from the sections. The slides were then placed in 100% I, 100% II, 95%, 80%, and 75% alcohol for 10 min each for hydration. Next, the slides were washed twice in double distilled water for 3 min each. Antigen retrieval was then performed with citrate buffer (pH 6.0) for 30 min, and the slides were naturally cooled to room temperature. The slides were then washed twice in double distilled water for 3 min each. Next, the slides were incubated with 5% BSA blocking solution for 1 h at room temperature and then washed thrice with PBS for 5 min each. The slides were then incubated with the primary antibody (PMI, 1:200 or ABCG2, 1:200) overnight at 4°C in a wet box. The following day, the slides were washed thrice with PBS for 5 min each. Then, fluorescent secondary antibody was added to the slides, and the slides were incubated for 1 h in a wet box, protected from light. This was followed by the addition of an anti-fluorescence quenching blocker containing DAPI to seal the slides. Finally, images of the stained sections were acquired using a laser confocal fluorescence microscope.

### Flow cytometric analysis of the cell cycle

After being attached to the wall, the cells were incubated with different treatments and then collected through centrifugation at 800 rpm for 5 min. The supernatant was then discarded, and the cell suspension was added to 70% ice-cold ethanol drop by drop for fixing overnight at 4°C; it was then resuspended with PBS, washed twice, and mixed with RNase and then heated in a water bath for 15 min at 37°C. The PI staining solution was then added. The suspension was kept away from light for 10 min and then analyzed by flow cytometry.

### Western blotting

Proteins were extracted using a whole protein extraction kit. The protein concentration was determined using the BCA method to calculate the loading volume. Then, 10% SDS-PAGE was prepared for protein separation at 120 V, and the gel was transferred to a polyvinylidene fluoride (PVDF) membrane at 300 mA for 2 h. The membrane was then blocked with rapid solution, washed thrice for 10 min each with TBST, and then incubated with a primary antibody overnight at 4°C. The antibody was recycled after washing the membrane thrice for 10 min each with TBST. The membrane was then incubated with horseradish peroxidase (HRP)-labeled secondary antibody at room temperature for 1 h. Then, the membrane was washed thrice with PBST for 10 min each. Finally, protein was detected using a chemiluminescence solution (ECL) and exposure to compression in a dark room. All results were analyzed using ImageJ.

### RT-PCR

Cells were seeded into six-well plates 1 day before experimentation, so that the next day’s apposition density was about 70%–80%. Two 1.5-mL sterile EP tubes were prepared, and 125 μL Opti-MEM was added to them. SiRNA was added to one tube, and lipo 3000 was added to the other. Thew medium in the six-well plates was then replaced with 1 mL of fresh complete medium. Then, the abovementioned mixture was evenly added dropwise into the wells of the six-well plate and mixed well through gentle shaking. The total cellular RNA was then extracted. Next, cDNA was obtained using the Tiangen reverse transcription kit. A negative control specimen and a positive quantitative reference specimen were created according to the manufacturer’s instructions of the Tiangen SYBR fluorescence quantification kit. A total of 20 μL of the total reaction was used to perform PCR experiments according to the Step one plus operating procedure. Each RT-PCR experiment was repeated thrice, and the relative levels of target gene expression were calculated and analyzed using the 2^−ΔΔT^ method (GAPDH was used as the internal reference gene).

### LBP active ingredient and target screening

Active component searches for LBP were performed using the Traditional Chinese Medicine Systematic Pharmacology Database and Analysis Platform (TCMSP, https://tcmspe.com/) according to the following screening criteria: drug-like properties (DL) ≥ 0.18 and oral bioavailability (OB) ≥ 30%. The active ingredient targets were screened using TCMSP, and the CAS numbers were entered in the organic small molecule bioactivity database (PubChem, https://pubchem.ncbi.nlm.nih.gov/) to obtain the SDF format of the 2D structures of the compound components. Then, the SDF format files of the compounds were imported into the Swiss Target Prediction database (http://www.swisstargetprediction.ch/) to obtain the predicted targets of each chemical component. These predicted targets were then filtered based on a probability >0 to obtain the active component targets. The chemical composition-related target information from the Swiss Target Prediction and TCMSP databases were integrated to obtain the potential targets of LBP.

### Acquisition of potential targets for LBP in CC treatment

The Gene Cards database (https://www.genecards.org/) was used to search the keyword “colon cancer.” The relevant disease targets with relevance score ≥10 were screened. Using the online Venn analysis tool (http://www.bioinformatics.com.cn/login/), the intersection of active ingredient targets and CC-related targets was identified in the Venn diagram. The obtained intersection targets were considered as the potential targets of LBP for CC treatment.

### PPI network analysis and key target acquisition

The potential targets of LBP in CC treatment were uploaded to the STRING database (https://string-db.org/) to obtain the interaction results. The results were then imported into the Cytoscape 3.9.1 software. After the PPI network was plotted, the top 30 core genes with the highest node degree were filtered out. The larger the node degree, the more important is the role of LBP in CC treatment.

### GO functional enrichment and KEGG pathway enrichment analyses

To further elaborate the mechanism underlying the therapeutic effect of LBP in CC, GO functional enrichment and KEGG pathway enrichment analyses were performed on the intersection targets identified using the R language. The relevant results were output in the form of bar graphs. *p* < 0.05 was considered to indicate a statistically significant difference.

### Molecular docking

To further clarify the intrinsic molecular mechanism of LBP-mediated CC treatment, the core targets were molecularly docked to the active LBP components and PMI. The protein structures of the core targets were downloaded from Uniprot (https://www.uniprot.org/). The protein receptors and small molecule ligands were routinely processed using the Pymol and AutoDock softwares for molecular docking and binding energy calculation, respectively. The Pymol software was used to visualize some of the results.

### Statistical analysis

All data are expressed as mean ± SD. The *t*-test was used for comparisons between two groups, and one-way analysis of variance was used for multi-group analyses. All images were created using the GraphPad Prism, Adobe Photoshop, and Adobe Illustrator softwares. *p* < 0.05 was considered statistically significant.

## Results

### Effect of *L. barbarum* polysaccharide combined with oxaliplatin on the invasion and migration abilities and cell cycle of HCT116-OXR cells

**FIGURE 1:**
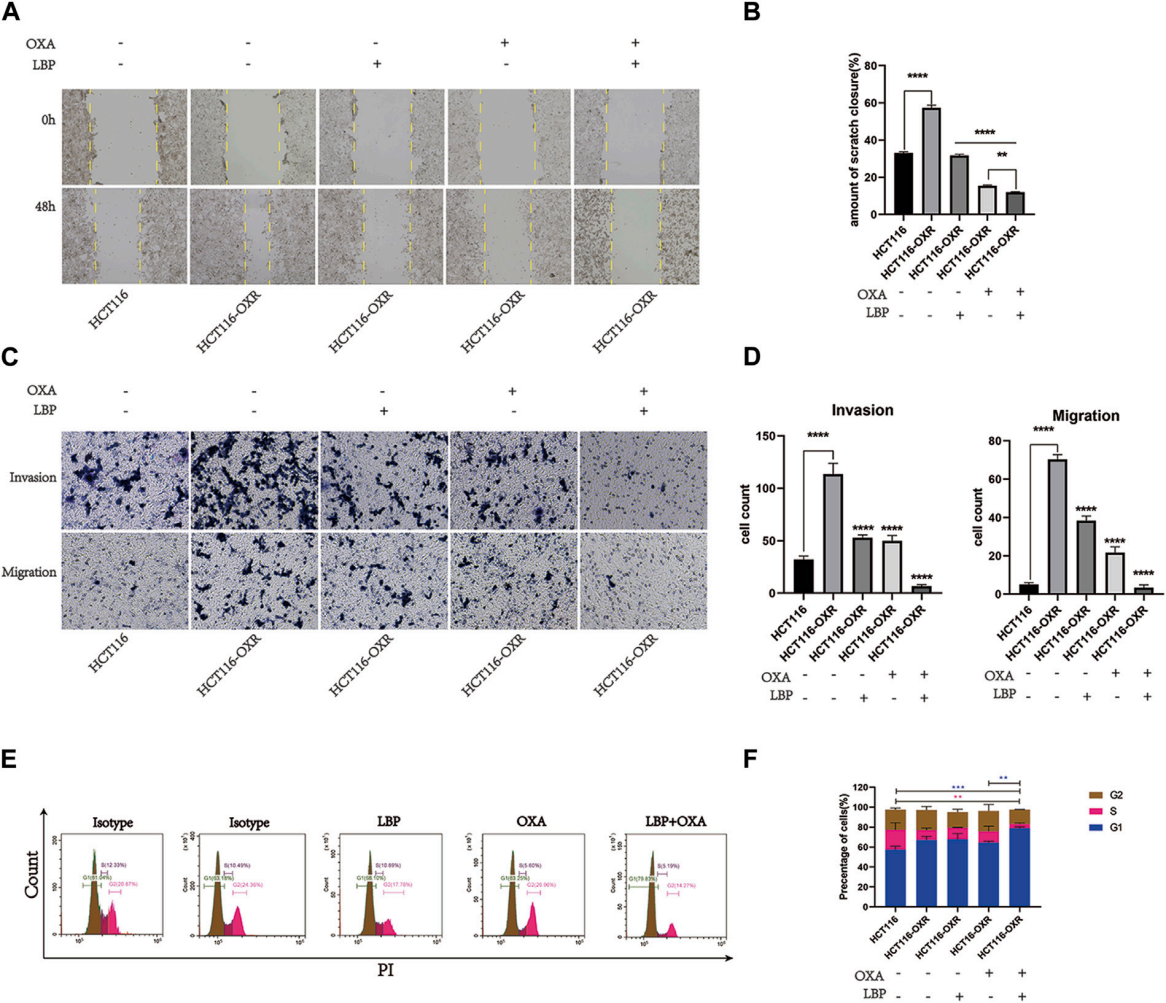
*Lycium barbarum* polysaccharide combined with oxaliplatin inhibited the invasion and migration abilities of HCT116-OXR cells. **(A)** Scratch test results; **(B)** Scratch healing rate in each group (*p < 0.05, **p < 0.01, ***p < 0.001, ****p < 0.0001). **(C)** Invasion and migration assay (100×) results. **(D)** The cell invasive and migration statistics for the HCT116 control group, HCT116-OXR blank treatment group, LBP treatment group, oxaliplatin treatment group, and LBP combined with oxaliplatin treatment group (*p < 0.05, **p < 0.01,***p < 0.001,****p < 0.0001). **(E)** Cell cycle analysis results for the HCT116 control group, HCT116-OXR blank treatment group, LBP treatment group, oxaliplatin treatment group, and LBP combined with oxaliplatin treatment group. **(F)** Proportions of cells in the G1, M, and G2 phases in all treatment groups.

The invasion and migration abilities of HCT116-OXR cells were enhanced (Figure 1) and the numbers of invading and migrating cells were significantly decreased after treatment with LBP combined with oxaliplatin (Figure 1). LBP combined with oxaliplatin significantly inhibited the invasion and migration abilities of HCT116-OXR cells. The proportions of cells in the G1 phase were 68.10%, 63.25%, and 79.83%, and the proportions of cells in the G2 phase were 17.76%, 26.96%, and 14.27% in the LBP treatment group, oxaliplatin treatment group, and LBP combined with oxaliplatin treatment group, respectively (Figure 1). Compared with the control group, the proportion of HCT116-OXR cells in the G1 phase increased from 63.18% to 79.83% and the proportion of HCT116-OXR cells in the G2 phase decreased from 24.36% to 14.27% after the treatment with LBP combined with oxaliplatin. These findings suggested that LBP combined with oxaliplatin significantly promoted G1 phase arrest (Figure 1), thereby inhibiting HCT116-OXR cell proliferation.

### PMI gene knockdown and inhibition of PI3K, AKT, Bcl-2, and Bax protein expression

**FIGURE 2:**
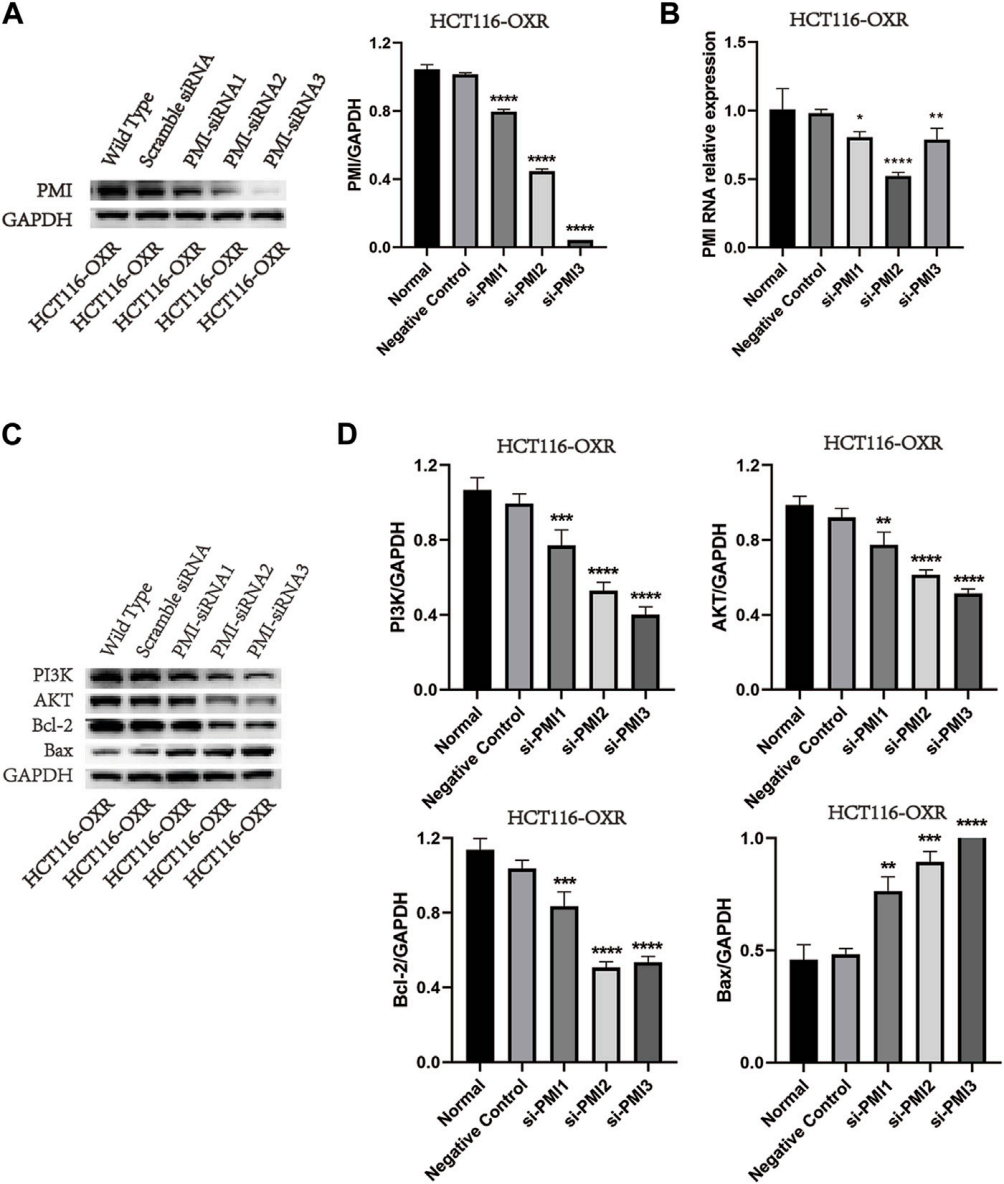
PMI gene knockdown and inhibition of PI3K, AKT, Bcl-2, and Bax protein expression levels. **(A)** Quantification of PMI expression (*n* = 3). **(B)** Statistical analysis of the PMI gene expression level (*n* = 3). **(C)** Quantification of PI3K, AKT, Bcl-2, and Bax protein expression levels. **(D)** Statistical analysis of PI3K, AKT, Bcl-2, and Bax protein expression levels (*n* = 3).

Knockdown of PMI expression significantly downregulated PMI expression in drug-resistant colon cancer cells (Figure 2). Compared with the negative treatment group, knockdown of PMI expression significantly downregulated the expression levels of PI3K, AKT, and Bcl-2 and upregulated the expression level of Bax (Figure 2), indicating that PMI gene knockdown inhibited the PI3K/AKT pathway (Figure 2).

### LBP inhibited tumor growth in BALB/c mice

**FIGURE 3:**
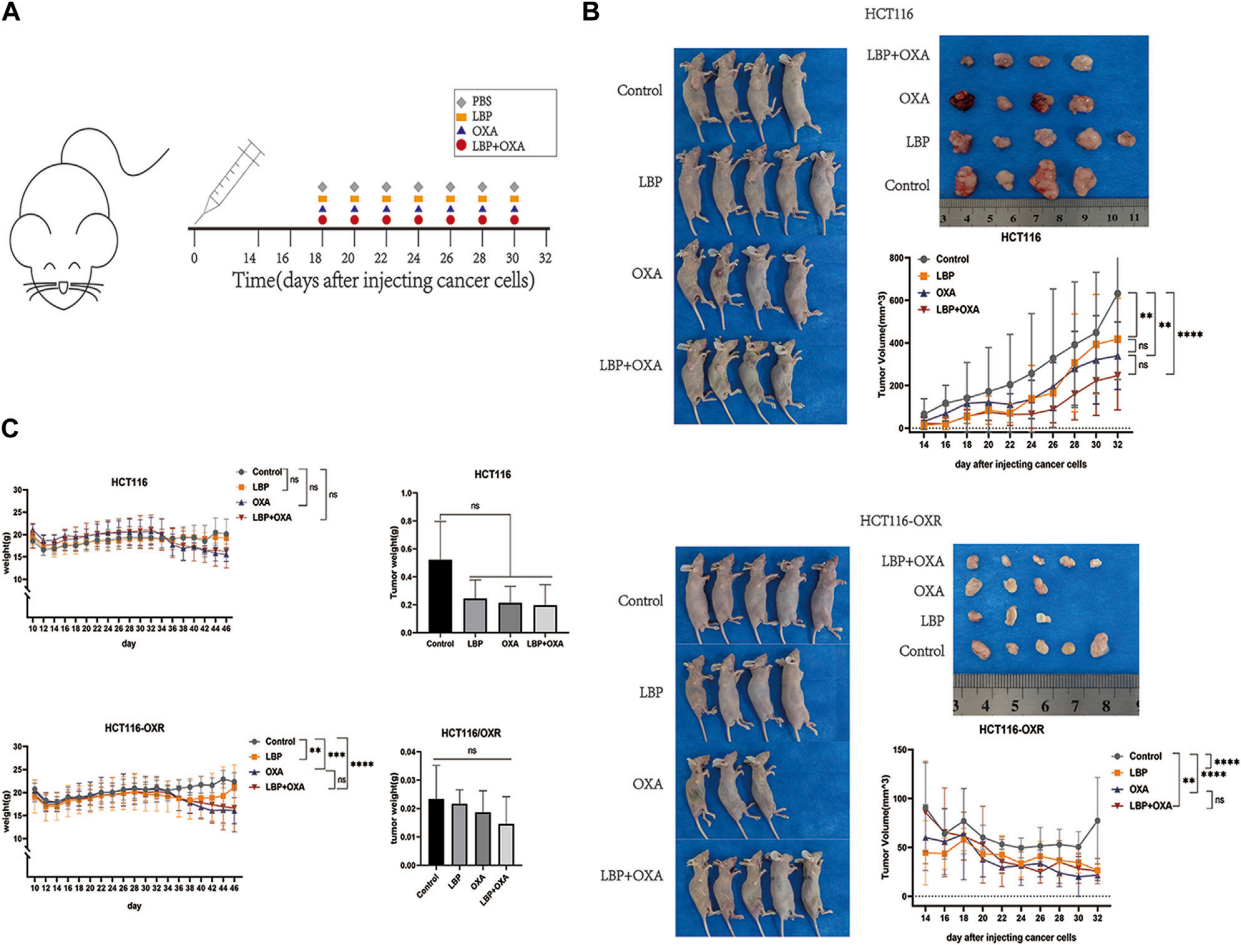
Effects of LBP on xenografted colon cancer model mice. **(A)** Schematic diagram of the experimental schedule. **(B)** Changes in tumor size over the 14 days of experimentation in all groups. Tumor volume = 1/2ab^2^. (*p < 0.05, **p < 0.01, ***p < 0.001, ****p < 0.0001). **(C)** Body and tumor weight changes over the 14 days of experimentation (*p < 0.05, **p < 0.01, ***p < 0.001, ****p < 0.0001).

The tumor volumes in the LBP groups, OXA groups, and LBP + OXA groups were all significantly lower than those in the blank treatment groups (Figure 3). However, there was no difference between the tumor volumes of the OXA groups and LBP + OXA groups (Figure 3B). There was also no difference between the tumor weights of various groups (Figure 3C).

### LBP showed no organ toxicity in BALB/c mice

**FIGURE 4:**
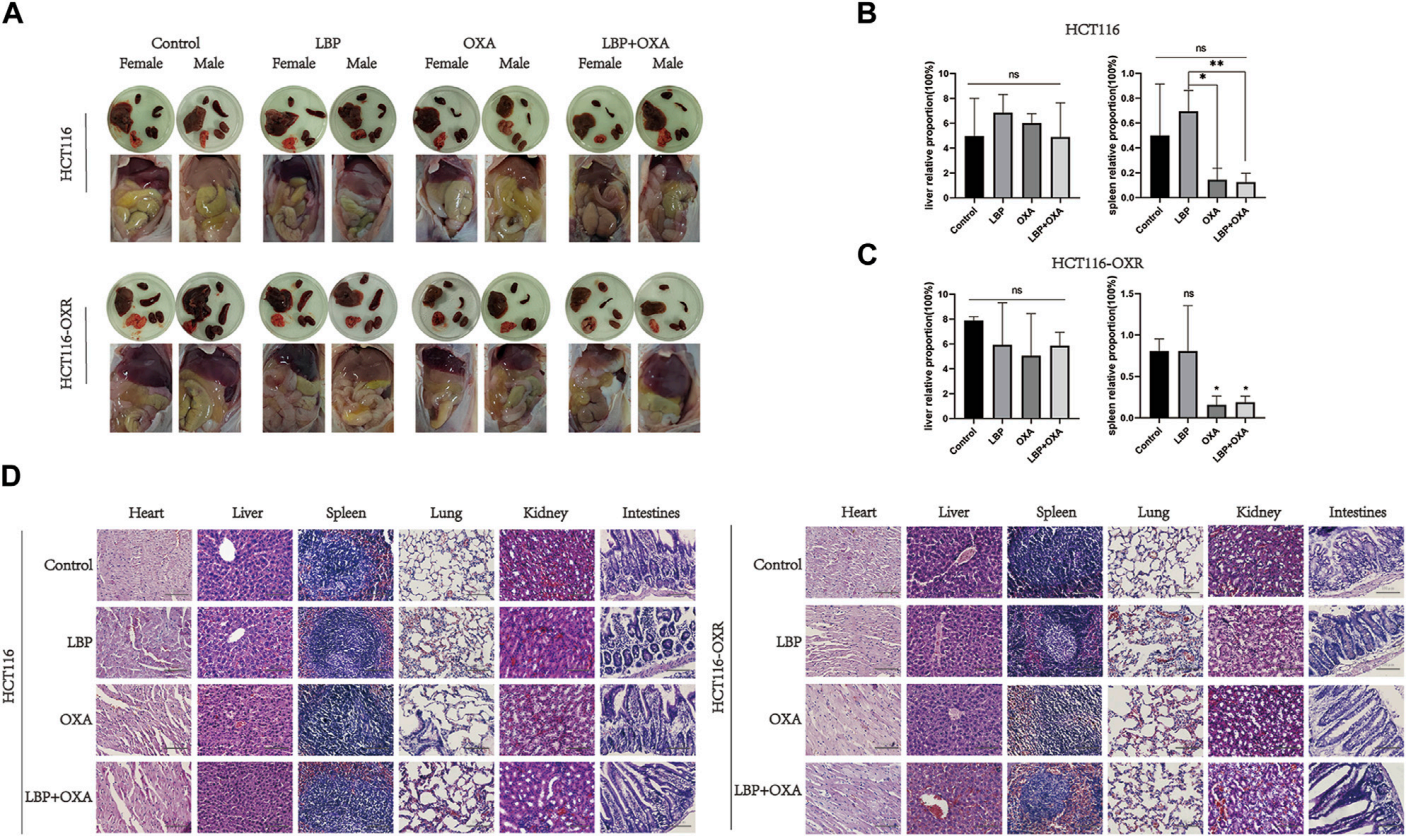
LBP showed no organ toxicity in BALB/c mice. **(A)** Visual inspection of the internal organs of xenografted HCT116 and HCT116-OXR colon cancer model mice in each group **(B and C)** was performed to determine the liver and spleen coefficients. Liver coefficient = liver weight/body weight × 100%, spleen coefficient = spleen weight/body weight × 100% (*p < 0.05, **p < 0.01, ***p < 0.001, ****p < 0.0001). **(D)** HE×100 staining results. A and B show the HE×100 staining results for the heart, liver, spleen, lung, kidney, and large intestine in HCT116 and HCT116-OXR model mice, respectively. Scale bar = 100 μm.

Visual inspection results for the internal organs of xenografted colon cancer model mice in each group are shown in Figure 4A. There was no significant difference between the liver coefficients of the groups (Figures 4A,B). Compared with the PBS treatment group, there was no significant difference in the spleen coefficients of other groups. In the HCT116 model mice, the spleen coefficients of the OXA group and LBP + OXA group were significantly lower compared with that of the LBP treatment group, possibly due to an oxaliplatin side effect on the spleen (Figure 4C). In the HCT116-OXR model mice, the spleen coefficients of the OXA group and LBP + OXA group were significantly lower compared with that of the blank treatment group, possibly due to an oxaliplatin side effect on the spleen. The H&E staining results are shown in Figure 4. Hematoxylin and eosin (H&E) stained the cell nucleic acids and proteins a deep blue-purple/pink color, making it possible to visualize cell structure and analyze cell morphology. The staining revealed no obvious nuclear atypia and no distant organ metastases (Figure 4D).

Immunohistochemistry revealed the PMI, ABCG2, PI3K, and AKT protein expression levels in each xenografted colon cancer model mouse group.

**FIGURE 5:**
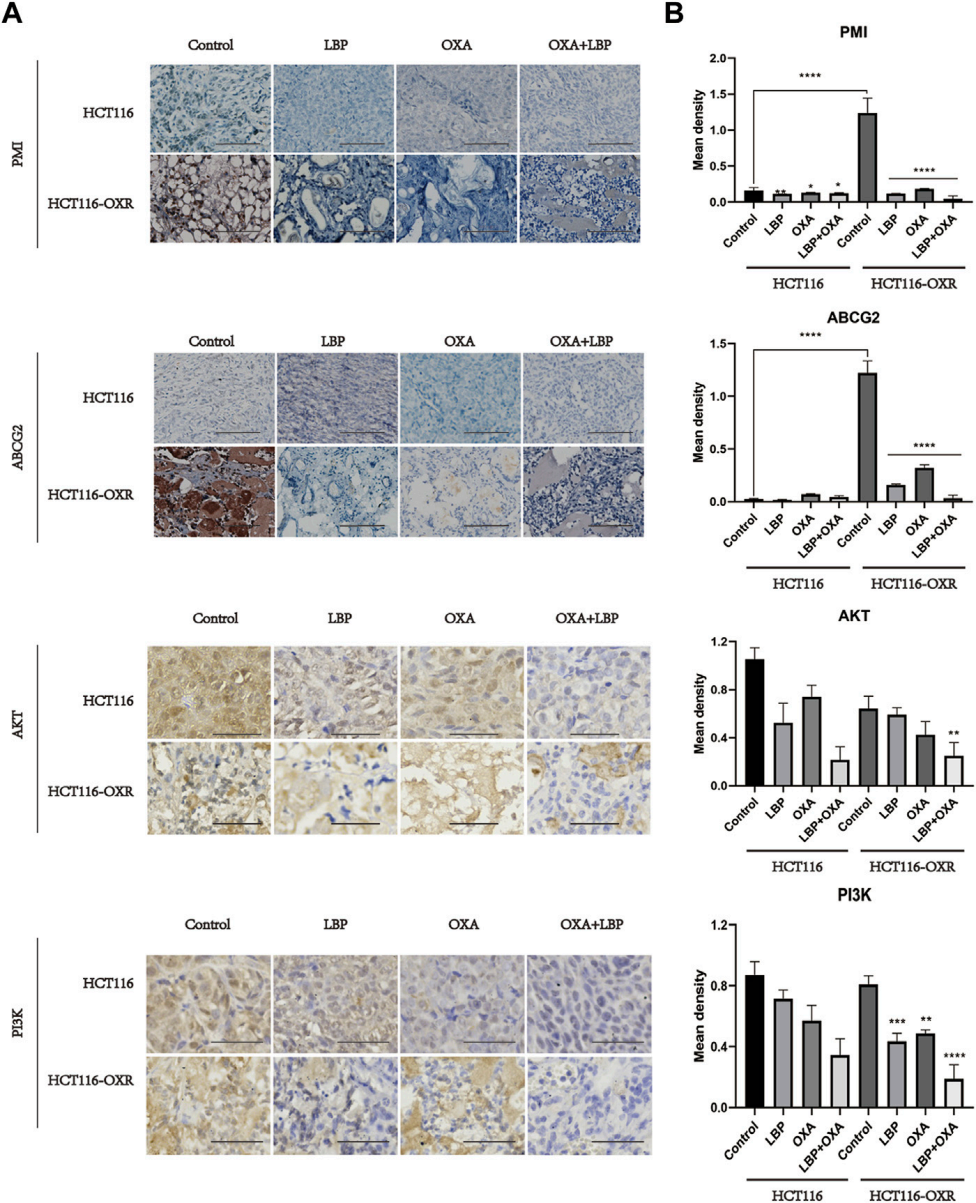
Immunohistochemistry revealed the PMI, ABCG2, PI3K, and AKT protein expression levels in each xenografted colon cancer model mouse group (IHC×100). **(A)** Immunohistochemical results for PMI, ABCG2, PI3K, and AKT in HCT116 and HCT116-OXR model mice. Scale bar = 100 μm. **(B)** Quantitative analysis of protein expression in each group. (*p < 0.05, **p < 0.01, ***p < 0.001, ****p < 0.0001).

The expression levels of PMI and ABCG2 in tumor tissues were determined using immunohistochemistry (Figure 5). The protein expression levels of PI3K and AKT decreased after treatment with LBP + OXA (Figure 5).

### Immunofluorescence, western blot, and co-localization analyses for PMI and ABCG2 expression

**FIGURE 6:**
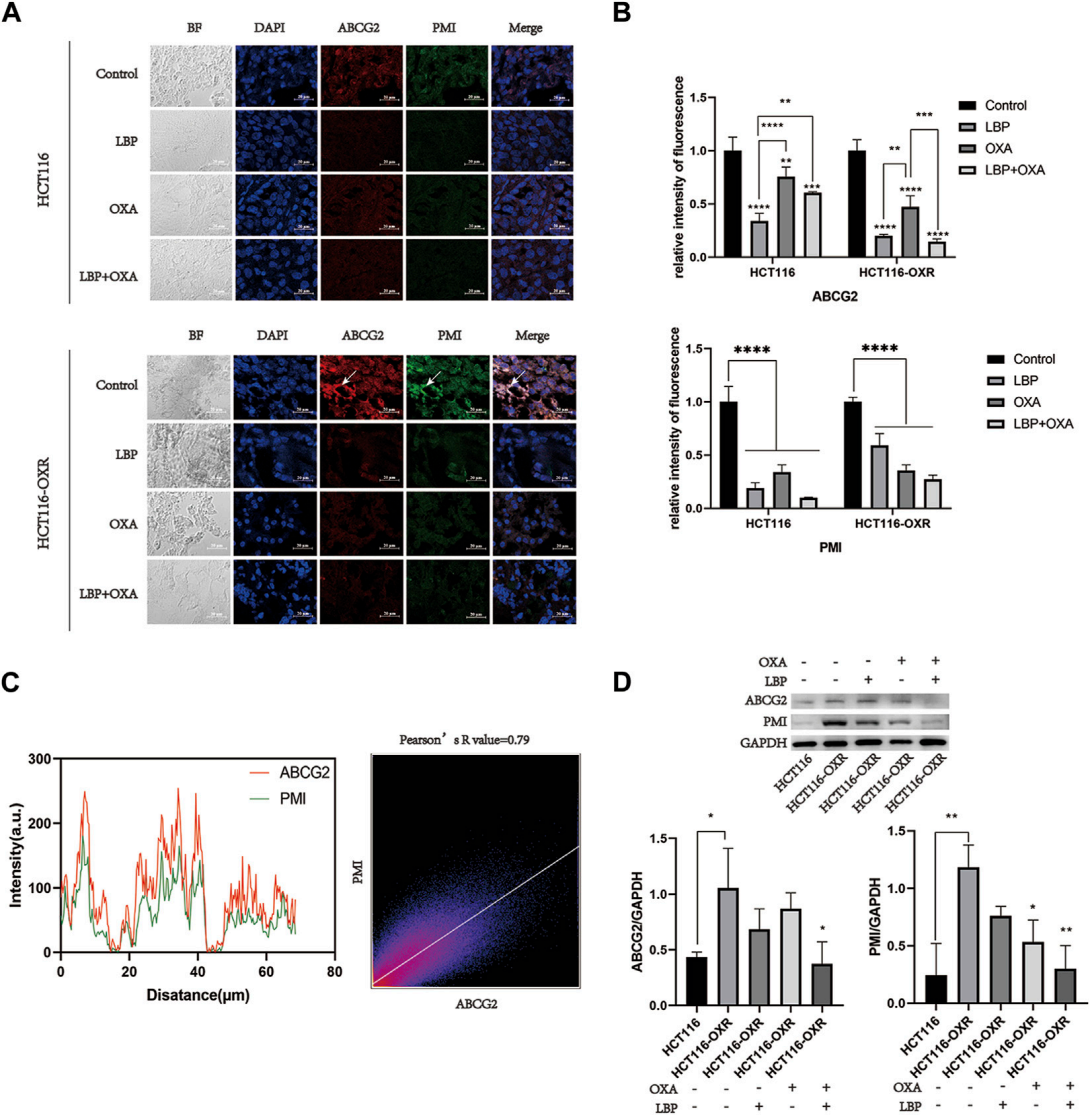
Immunofluorescence, western blot, and co-localization analysis results for PMI and ABCG2 expression in tumor tissues of model mice. **(A)** Immunofluorescence analysis revealed PMI (green) and ABCG2 (red) expression (400×). Arrows indicate high expression. Scale bar = 20 μm. **(B)** Quantitative analysis of fluorescence intensity (*p < 0.05, **p < 0.01, ***p < 0.001, ****p < 0.0001). **(C)** Colocalization analysis results for PMI and ABCG2. **(D)** Western blotting results for PMI and ABCG2 expression and their quantitative analysis in the HCT116 blank treatment group, HCT116-OXR blank treatment group, HCT116-OXR LBP group, OXA group, and LBP + OXA group (*p < 0.05, **p < 0.01, ***p < 0.001, ****p < 0.0001).

The expression of PMI and ABCG2 in tumor tissues was assessed using immunofluorescence and western blot analyses. PMI and ABCG2 expression levels decreased after LBP + OXA treatment (Figures 6A,B,D). The colocalization analysis showed that high PMI expression was likely to be accompanied by high ABCG2 expression, with the Pearson’s R value being 0.79 (Figure 6C).

### Screening of LBP active components

The TCMSP database was used to screen using the OB ≥ 30% and DL ≥ 0.18 criteria, and 45 active components, including carotenoids, phenylpropanoids, quercetin, and xanthophylls, were identified (Table 1).

**TABLE 1:** Active ingredients.

MOL ID	MOL name	MW	OB(%)	DL
MOL010234	delta-Carotene	536.96	31.8	0.55
MOL009681	Obtusifoliol	426.8	42.55	0.76
MOL009678	lanost-8-enol	428.82	34.23	0.74
MOL009677	lanost-8-en-3beta-ol	428.82	34.23	0.74
MOL009665	Physcion-8-O-beta-D-gentiobioside	608.6	43.9	0.62
MOL009664	Physalin A	526.58	91.71	0.27
MOL009662	Lantadene A	552.87	38.68	0.57
MOL009660	methyl (1R,4aS,7R,7aS)-4a,7-dihydroxy-7-methyl-1-[(2S,3R,4S,5S,6R)-3,4,5-trihydroxy-6-(hydroxymethyl)oxan-2-yl] oxy-1,5,6,7a-tetrahydrocyclopenta [d]pyran-4-carboxylate	406.43	39.43	0.47
MOL009656	(E,E)-1-ethyl octadeca-3,13-dienoate	308.56	42	0.19
MOL009653	Cycloeucalenol	426.8	39.73	0.79
MOL009651	Cryptoxanthin monoepoxide	568.96	46.95	0.56
MOL009650	Atropine	289.41	42.16	0.19
MOL009646	7-O-Methylluteolin-6-C-beta-glucoside_qt	318.3	40.77	0.3
MOL009644	6-Fluoroindole-7-Dehydrocholesterol	402.7	43.73	0.72
MOL009642	4alpha-methyl-24-ethylcholesta-7,24-dienol	426.8	42.3	0.78
MOL009641	4alpha,24-dimethylcholesta-7,24-dienol	412.77	42.65	0.75
MOL009640	4alpha,14alpha,24-trimethylcholesta-8,24-dienol	426.8	38.91	0.76
MOL009639	Lophenol	400.76	38.13	0.71
MOL009635	4,24-methyllophenol	414.79	37.83	0.75
MOL009634	31-norlanosterol	412.77	42.2	0.73
MOL009633	31-norlanost-9 (11)-enol	414.79	38.35	0.72
MOL009631	31-Norcyclolaudenol	440.83	38.68	0.81
MOL009622	Fucosterol	412.77	43.78	0.76
MOL009621	24-methylenelanost-8-enol	440.83	42.37	0.77
MOL009620	24-methyl-31-norlanost-9 (11)-enol	428.82	38	0.75
MOL009618	24-ethylcholesta-5,22-dienol	412.77	43.83	0.76
MOL009617	24-ethylcholest-22-enol	414.79	37.09	0.75
MOL009615	24-Methylenecycloartan-3beta,21-diol	456.83	37.32	0.8
MOL009612	(24R)-4alpha-Methyl-24-ethylcholesta-7,25-dien-3beta-ylacetate	482.87	46.36	0.84
MOL009604	14b-pregnane	288.57	34.78	0.34
MOL008400	glycitein	284.28	50.48	0.24
MOL008173	daucosterol_qt	414.79	36.91	0.75
MOL007449	24-methylidenelophenol	412.77	44.19	0.75
MOL006209	cyanin	411.66	47.42	0.76
MOL005438	campesterol	400.76	37.58	0.71
MOL005406	atropine	289.41	45.97	0.19
MOL003578	Cycloartenol	426.8	38.69	0.78
MOL001979	LAN	426.8	42.12	0.75
MOL001495	Ethyl linolenate	306.54	46.1	0.2
MOL001494	Mandenol	308.56	42	0.19
MOL001323	Sitosterol alpha1	426.8	43.28	0.78
MOL000953	CLR	386.73	37.87	0.68
MOL000449	Stigmasterol	412.77	43.83	0.76
MOL000358	beta-sitosterol	414.79	36.91	0.75
MOL000098	quercetin	302.25	46.43	0.28

### Construction of a PPI network and identification of core proteins

**FIGURE 7:**
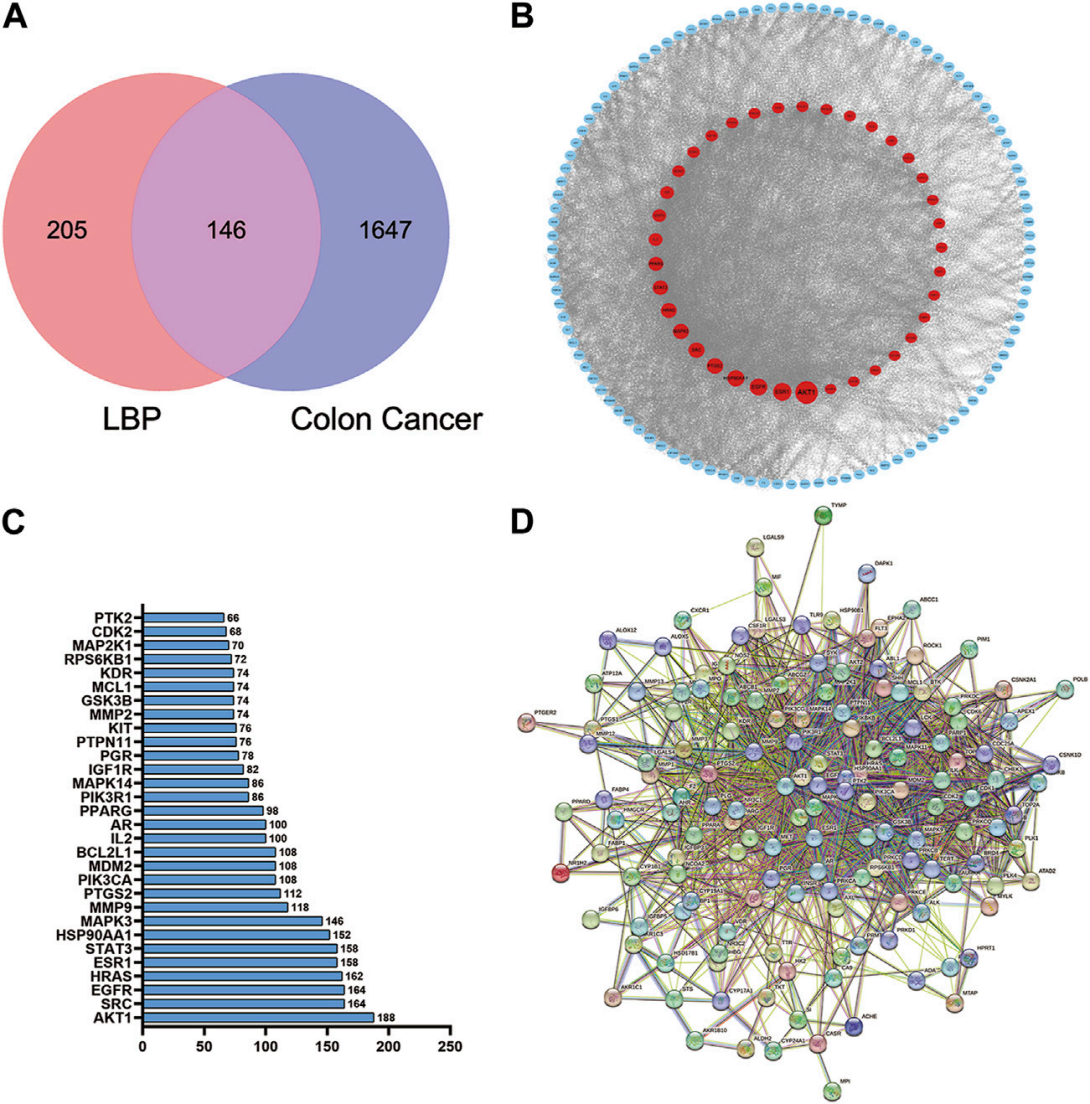
Venn diagram of the LBP and CC intersection targets and PPI network of core proteins. **(A)** Venn diagram of the LBP and CC intersection targets. **(B)** PPI network of LBP targets in CC treatment. **(C)** PPI plot ranking the top 30 intersection targets. **(D)** PPI plot of PMI with the 146 potential targets.

A total of 351 herbal ingredient targets were retrieved from the TCMSP database and Swiss Target Prediction database. A total of 1793 CC-related target genes were obtained from the GeneCards database. Using an online Venn analysis tool, the targets of LBP and CC were intersected to obtain 146 potential targets for the treatment of CC with LBP (Figure 7). The interactions of potential LBP targets in CC treatment were obtained from the STRING database and imported into Cytoscape 3.9.1 for visualization (Figure 7). The 146 potential targets were included in the network with node degree as the evaluation parameter; the higher is the node degree, the more important it is in the network, the more are the targets connected to the node. The top 30 targets in the PPI network in terms of node degree are shown in Figure 7; among these, the top five core targets in terms of degree were AKT1, SRC, EGFR, HRAS, and ESR1. The interactions between PMI and the 146 potential targets were retrieved using STRING (Figure 7).

### GO functional enrichment and KEGG pathway enrichment analyses

**FIGURE 8:**
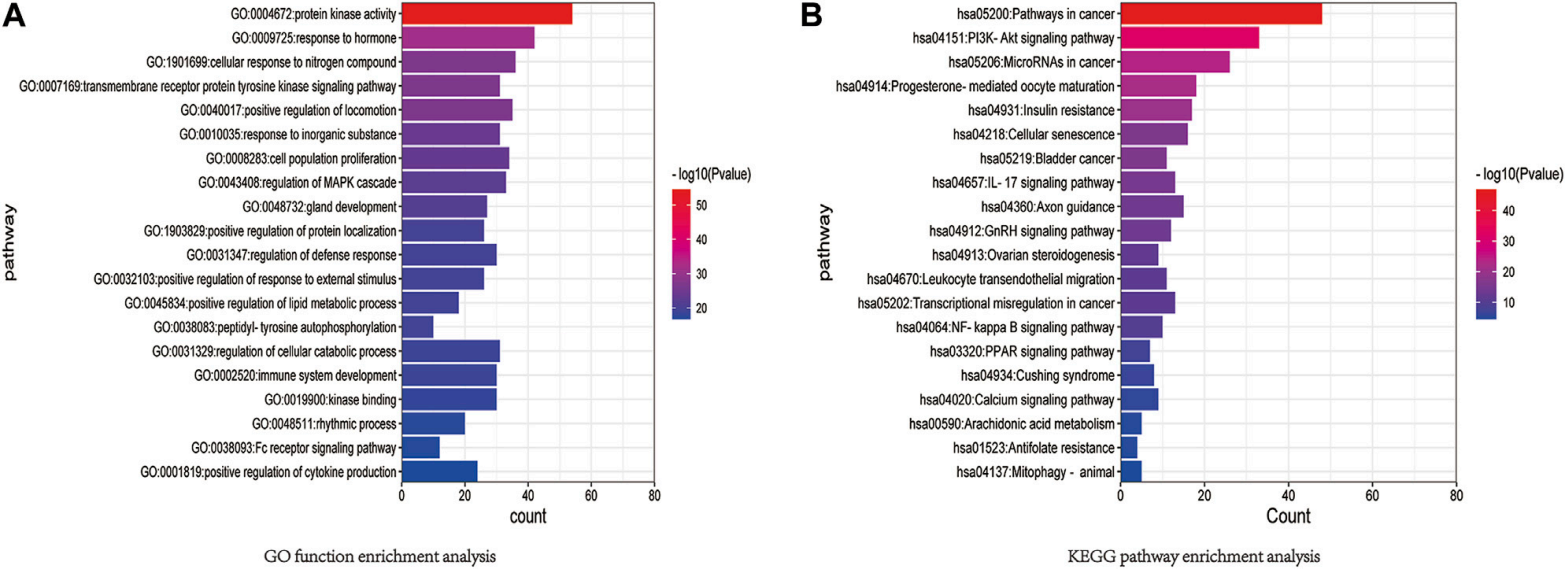
**(A)** GO function enrichment analysis. **(B)** KEGG pathway enrichment analysis.

The results of GO functional enrichment and KEGG pathway enrichment (*p <* 0.05) for the 146 potential targets were analyzed. GO functional enrichment results mainly involved protein kinase activity, hormone response, cellular response to nitrogen compounds, tyrosine kinase signaling proteins, motor positive regulatory proteins, inorganic substance response, cell population proliferation proteins, etc. (Figure 8). KEGG pathway enrichment results mainly involved cancer-related signaling pathways, PI3K-Akt signaling pathway, cancer microRNAs, progesterone-mediated follicle maturation pathway, insulin resistance pathway, cellular senescence signaling pathway, bladder cancer signaling pathway, IL-17 signaling pathways, axon guidance signaling pathway, and gonadotropin-releasing hormone signaling pathway (Figure 8). Among these, the PI3K/AKT signaling pathway plays an important role in the mechanism of drug resistance in colon cancer.

### Molecular docking analysis

**FIGURE 9:**
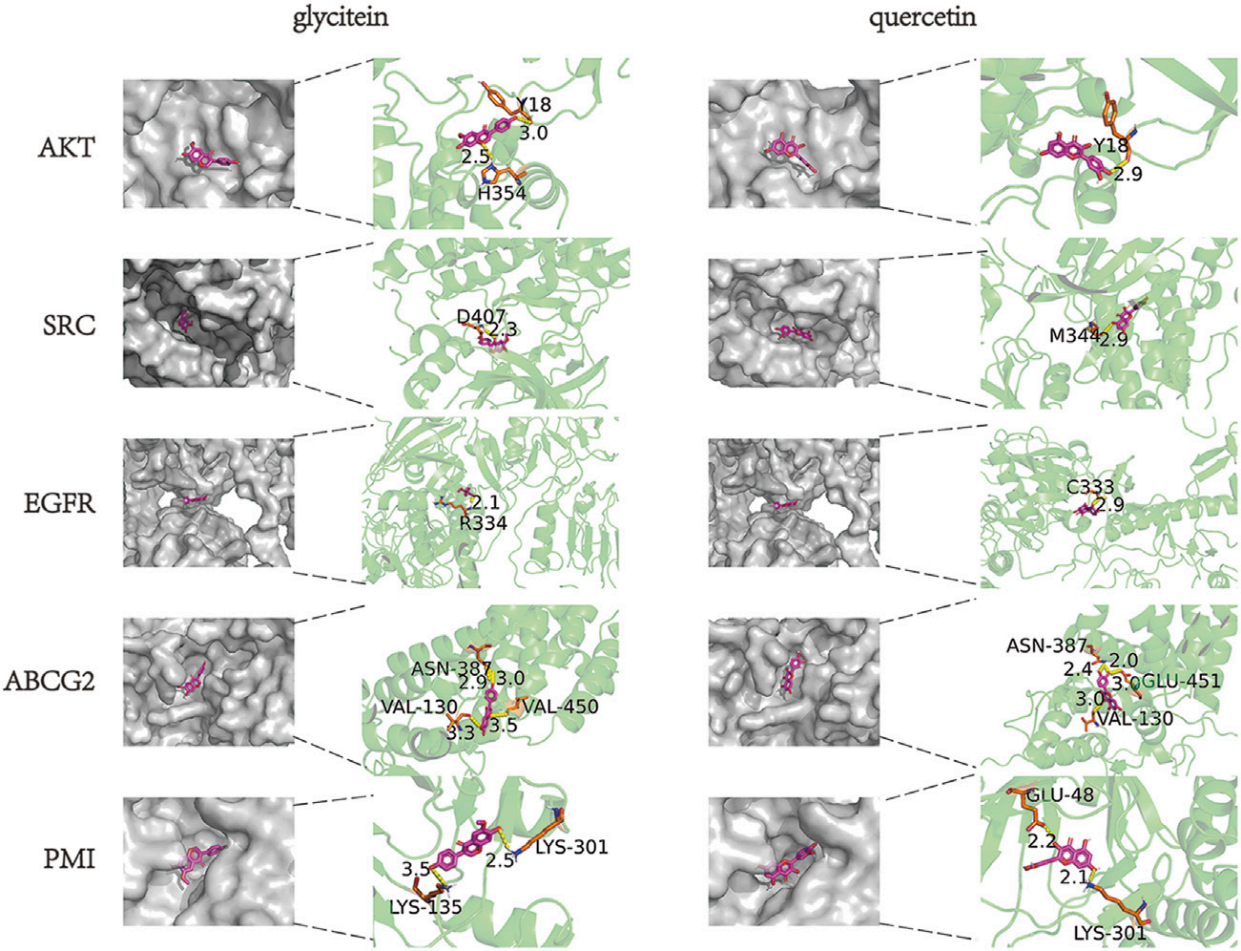
Molecular docking of LBP active components to potential CC targets.

In order to clarify the binding efficacy between the key targets of LBP active components in CC and predict the possible binding sites between LBP and PMI, two core components of the drug-component-target-disease modulation network, quercetin and glycitein, were selected and molecularly docked with the top-ranked core targets and PMI in the PPI network (Figure 9). Usually, binding energies less than 0 indicate possible binding between molecules, and binding energies less than—4.25 kcal/mol^−1^ indicate certain binding between molecules; that is, smaller binding energy values indicate stronger binding abilities. After docking the two active components, quercetin and glycitein, with CC target proteins, it was found that the flavonoids had strong binding ability to SRC, EGFR, ABCG2, and PMI (Table 2).

**TABLE 2:** Scoring of docking binding energy of key compounds to target molecules (kcal/mol).

Compound	AKT	SRC	ESR1	EGFR	HRAS	ABCG2	PMI
glycitein	−3.04	−5.36	−3.59	−4.34	−2.52	−4.75	−4.35
quercetin	−2.23	−2.96	−4.5	−3.08	−3.37	−4.12	−2.11

## Discussion

With the increase in the global cancer burden, cancer has become the second leading cause of death worldwide, after heart disease and colorectal cancer, in terms of both cancer incidence and cancer-associated mortality in both men and women (Siegel et al., 2023). Eighty percent of colon cancer patients are ≥60 years old at diagnosis. The most common cause of colorectal cancer is Lynch syndrome, followed by familial adenomatous polyposis; chronic colitis caused by inflammatory bowel disease (IBD) is also associated with an increased risk of colorectal cancer (Kuipers et al., 2015).

Scholars advocate molecular staging, such as through immunohistochemistry, for all colon cancers as well as detection of mismatch repair defects (MMR-D) or microsatellite instability high (MSI-H) through polymerase chain reaction to screen for Lynch syndrome and mutations in KRAS, NRAS, and BRAF in metastatic colorectal cancer (Biller and Schrag, 2021). For 50% of patients with KRAS/NRAS/BRAF wild-type metastatic colorectal cancer, the median survival after treatment is approximately 30 months (Cremolini et al., 2015). The activity of protein kinases encompasses transmembrane receptor protein kinase activity, protein tyrosine kinase activity, protein serine/threonine kinase activity, and cyclin-dependent protein kinase activity. For instance, one example of a protein with kinase activity is the protein kinase C (PKC). Colon carcinogenesis involves a complex series of events that lead to progressive alterations in signaling pathways governing colonic epithelial cell proliferation, differentiation, and survival. It has been extensively documented that colon carcinogenesis in both rodents and humans is accompanied by specific changes in the expression patterns of PKC isozymes. The median overall survival of 80% of patients was about 1 year, that of 40% was 3 years, and that of 20% was 5 years after receiving first-line chemotherapy (Venook et al., 2017). The most significant issue with the use of multiple clinical chemotherapy regimens is tumor resistance and recurrence, which negatively impact medication efficacy. Therefore, the discovery of novel medications is necessary to address this issue. Plant polysaccharide has demonstrated impressive anti-cancer properties in earlier investigations (Zhang et al., 2013; Jiao et al., 2016). The synergistic effect of plant polysaccharide-based drugs and chemotherapeutic agents is significant. Studies have shown that fucose has the potential to increase the efficiency of drug delivery to tumors and achieve synergistic effects with other anti-cancer drugs (Reyes et al., 2020). The role of glycans in the development of multi-drug resistance in cancers can be attributed to abnormal functional aspects of glycolysis and glycolysis-related enzymes, the glycan structure, and glycan-forming process-related enzymes and glycoproteins (Wang et al., 2021).

*Lycium barbarum* polysaccharide is extracted from the *L. barbarum* L., a member of the nightshade family. Polysaccharides are found in well-known herbal formulations and have been used in China for over 2300 years (Jin et al., 2013). They have also been widely used as a dietary supplement to boost the body’s defense system and control blood pressure and blood sugar (Yang et al., 2022). An increasing number of studies have confirmed the anti-tumor effect of LBP (Luo et al., 2009; Shen and Du, 2012; Zhu and Zhang, 2013). By boosting the immune system, polysaccharide-protein complexes also exert inhibitory effects on S180 cells. For example, they increase macrophage phagocytosis, splenic lymphocyte proliferation, antibody secretion from splenocytes, cytotoxic T lymphocyte activity, and IL-2 mRNA expression while decreasing lipid peroxidation (Gan et al., 2004). LBP also has anti-tumor and chemo-protective properties that may lessen Adriamycin’s immunotoxicity and boost its anti-hepatocarcinogenic effects (Han et al., 2022). In the present study, network pharmacology results predicted that the active components of LBP had 146 common core targets with colon cancer. KEGG pathway enrichment analysis revealed the enrichment of multiple pathways affected by LBP in CC. Molecular docking predicted the binding energies for the binding of LBP active components with the potential CC targets in CC. LBP combined with OXA inhibited the invasion and migration abilities of HCT116-OXR cells, and halted the cell cycle in the G1 phase. Knockdown of PMI significantly downregulated the expression levels of PI3K, AKT, and Bcl-2 and upregulated the expression level of Bax, indicating that PMI knockdown inhibited the PI3K/AKT pathway. *In vivo* experiments confirmed that LBP combined with oxaliplatin effectively inhibited tumor growth and did not affect the liver and spleen coefficients. These findings indicate that LBP has no organ toxicity and safely and effectively inhibits tumor growth.

Besides *L. barbarum*, natural products have been widely reported to have a broad spectrum of human health. Quercetin alone or combined with sorafenib downregulated key inflammatory, proliferative and angiogenesis-related genes (TNF-α, VEGF, P53 and NF-κB). Combined quercetin/sorafenib treatment markedly improved the morphology of the induced liver damage and showed significant antioxidant and anti-tumor effects. The advantage of combined treatment efficacy reported here can be attributed to quercetin’s prominent effects in modulating cell cycle arrest, apoptosis, oxidative stress and inflammation (Abdu et al., 2022a). Crocin efficiently improved the induced inflammation, oxidation, and liver damage parameters. The combination of crocin with sorafenib improved physiological parameters such as oxidation, inflammation and liver damage as compared to sorafenib alone. Through the improvement of inflammation and oxidation parameters and an anti-proliferative action, crocin improves the action of sorafenib (Abdu et al., 2022b). Steroidal saponins are a group of naturally occurring compounds that primarily exist as secondary metabolites in plant species. Recent studies have suggested that steroidal saponins possess significant anti-cancer capabilities (Bouabdallah et al., 2023). Natural products reverse drug resistance has been shown to have anti-tumor effects (Bharathiraja et al., 2023; Wu et al., 2023). So natural products and their derived biomolecules are potential resources to mine for novel anti-cancer drugs.

In the present study, the active components, potential targets, and signaling pathways of *L. barbarum* polysaccharide in colon cancer were predicted by network pharmacology combined with bioinformatics. Binding energies were predicted by molecular docking. The results of the GO functional enrichment analysis mainly included protein kinase activity, hormone response, cellular response to nitrogen compounds, tyrosine kinase signaling proteins, motor positive regulatory proteins, inorganic substance response, cellular response to nitrogen compounds, and inorganic substance response. Cellular response to nitrogen compounds refers to any cellular process that induces a change in the state or activity of a cell, such as movement, secretion, enzyme production, or gene expression, in response to stimulation by nitrogen compounds. The results of the KEGG pathway enrichment analysis mainly involved cancer-related signaling pathways, PI3K-Akt signaling pathway, cancer microRNAs, progesterone-mediated follicle maturation pathway, insulin resistance pathway, cellular senescence signaling pathway, bladder cancer signaling pathway, IL-17 signaling pathways, axon guidance signaling pathway, and gonadotropin-releasing hormone signaling pathway. The AKT protein family plays an important role in the functionality of the ABC family of drug-resistance proteins (Lu et al., 2020; Narayanan et al., 2021). The reversal of the dual functions of ABC-transporter-mediated and AKT-activation-enhanced MDR promises to improve current strategies based on combined drug treatments to overcome MDR challenges (Zhang L. et al., 2020). The EGFR family also occupies an important position in drug resistance-related signaling pathways. EGFR, also known as ERBB1 and HER1, is a transmembrane tyrosine kinase receptor (RTK). It is a member of the human epidermal receptor (HER) family and an important component of the cellular signaling pathway, and the upregulation of both EGFR gene and protein expression levels activate downstream pathways that are associated with cancer progression (Huang and Fu, 2015). The molecular docking results of the present study showed that SRC, EGFR, PMI, and ABCG2 had higher binding ability to the potential CC targets. The active components of LBP can thus be used to treat colon cancer by modulating numerous targets, including the identified core targets.

The high expression levels of PMI and ABCG2 in drug-resistant tissues of colon cancer were confirmed using immunohistochemistry, immunofluorescence, and western blot analyses. These levels were downregulated after treatment with LBP combined with oxaliplatin. Considering the important role of PMI in glycolysis and glycosylation (Ichikawa et al., 2014), previous antitumor studies have mostly focused on the role of PMI in glycolysis and glycosylation. In two such studies, PMI downregulation inhibited glycolysis and oxidative phosphorylation, thus suppressing tumor growth (Gonzalez et al., 2018; Saito et al., 2021). Another study showed that knockdown of PMI modulated fibroblast growth factor receptor (FGFR) family signaling and increased glioma radiosensitivity. Loss of PMI activity reduced phosphorylation of FGFR family receptors in U-251 and SKMG-3 malignant glioma cell lines and also led to significant reductions in FRS2, Akt, and MAPK signaling (Aurélie et al., 2014). Another study reported that PMI deletion can induce P53 expression, revealing a new possible mechanism to regulate P53 expression (Shtraizent et al., 2017). In the present study, the PI3K/AKT pathway was also downregulated after PMI knockdown, confirming our speculation. Therefore, drugs that effectively inhibit PMI have the potential to play a great role in cancer therapy. LBP can effectively inhibit the expression of the PMI/PI3K/AKT pathway, providing a new avenue for treating drug-resistant colon cancer.

The causes of resistance to clinical chemotherapy are complex and varied, and the “seed” theory, in which ABCG2 plays an important role, has received much attention for colon cancer. It has reported that natural product extract might suppress ABCG2-mediated MDR in colorectal cancer through inhibiting NF-kB signaling pathway (Sui et al., 2016). The results of the present study suggest that LBP reverses drug resistance in colon cancer cells by reducing ABCG2 expression. Most colorectal cancer cells originate from stem cells or stem cell-like cells at the base of the colonic crypt (Zeki et al., 2011). Mutations in oncogenes and tumor suppressor genes in these cells lead to the formation of cancer stem cells, which are essential for tumor initiation and progression. Mutations in some of these oncogenes and tumor suppressor genes, play an important role in the progression of colon cancer (Li et al., 2014). In turn, they show differences at the molecular level, and tumor cells show functional heterogeneity, which is also the theoretical basis of tumor stem cells. Tumor stem cell (CSC) pluripotency markers include CD133, ABCG2, and ALDH1A1 (An and Ongkeko, 2009). Drug-resistant cells exhibit high stem cell-like properties and highly express such stem cell markers. Current approaches to eradicate the drug-resistant cancer cell population include differentiation of CSCs, targeting of drug efflux proteins and other CSC surface markers, and inhibiting signaling pathways that maintain CSCs (Yang et al., 2020). The high expression of drug efflux proteins in drug-resistant colon cancer cells is one of the causes of their drug resistance. Additionally, it has been reported that drug-resistant colon cancer cells highly express the anti-apoptotic proteins Bcl-2, Bcl-XL, and Mcl-2, and have low expression of pro-apoptotic proteins, such as P53, Bax, and Bim; Bcl-2 family proteins play an important role in the drug resistance of colon cancer cells (Fulda, 2009; Hu et al., 2016). LBP combined with oxaliplatin inhibits ABCG2 expression, downregulates Bcl-2 expression, and upregulates Bax expression to promote apoptosis and thereby reverse drug resistance.

Moreover, our immunofluorescence results showed a statistical correlation of 79% for the co-localization of PMI and ABCG2, suggesting that PMI may play a role in the treatment of colon cancer using LBP by modulating ABCG2. The N596 site is the only site for the N-linked glycosylation of the ABCG2 protein that has been detected so far (Diop and Hrycyna, 2005; Mohrmann et al., 2005). However, N-linked glycosylation does not affect the expression and translocation of ABCG2 to the plasma membrane; therefore, it is not important for its overall function. N-linked glycosylation is important for ABCG2 stability, because the disruption of N-linked glycosylation leads to enhanced protein instability and ubiquitin-mediated proteasomal degradation (Nakagawa et al., 2009; Nakagawa et al., 2011). The role played by N-linked glycosylation in the formation of disulfide bonds between ABCG2 molecules and its importance for proper folding and enhancing the stability of human ABCG2 protein dimers in the endoplasmic reticulum have also been confirmed (Nakagawa et al., 2011). In the present study, LBP combined with oxaliplatin simultaneously inhibited the expression of PMI and ABCG2 and also confirmed the important role of PMI in the process of drug resistance in colon cancer. Given the important role of ABCG2 in stem cell formation and drug resistance in colon cancer, effects of changes in its expression deserve to be explored.

In summary, this study confirmed that LBP combined with oxaliplatin plays a role in reversing drug resistance in colon cancer cells by down-regulating the PMI/PI3K/AKT pathway and simultaneously inhibiting the expression of ABCG2^+^ colon cancer stem cells to promote apoptosis. Moreover, our findings suggest that LBP has no significant organ toxicity and is an ideal antitumor drug, laying the foundation for more in-depth studies aimed at its use in the clinical treatment of drug-resistant colon cancer in the future.

## Data Availability

The original contributions presented in the study are included in the article/Supplementary Materials, further inquiries can be directed to the corresponding authors.
