# Supplementary material for: Lycium barbarum polysaccharide reverses drug resistance in oxaliplatin-resistant colon cancer cells by inhibiting PI3K/AKT-dependent phosphomannose isomerase
Source: Front Pharmacol. 2024 Mar 21;15:1367747. doi: 10.3389/fphar.2024.1367747 (PMC10991850; doi:10.3389/fphar.2024.1367747)
Supplement: Supplementary file 1 [file DataSheet1.PDF]

1. In vitro

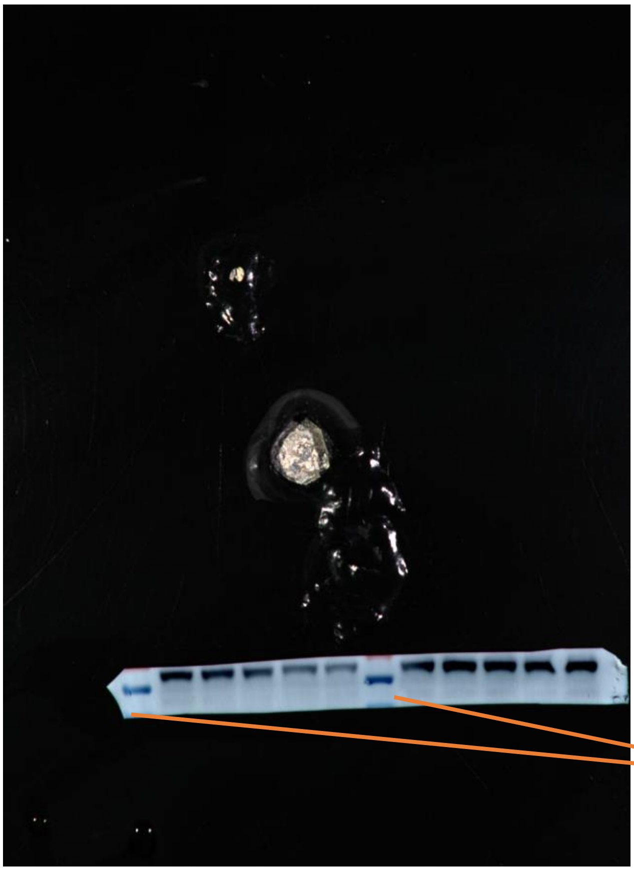
AKT(60KDa)
Marker(LOT:#26617,red means 70KDa, green means 10KDa)

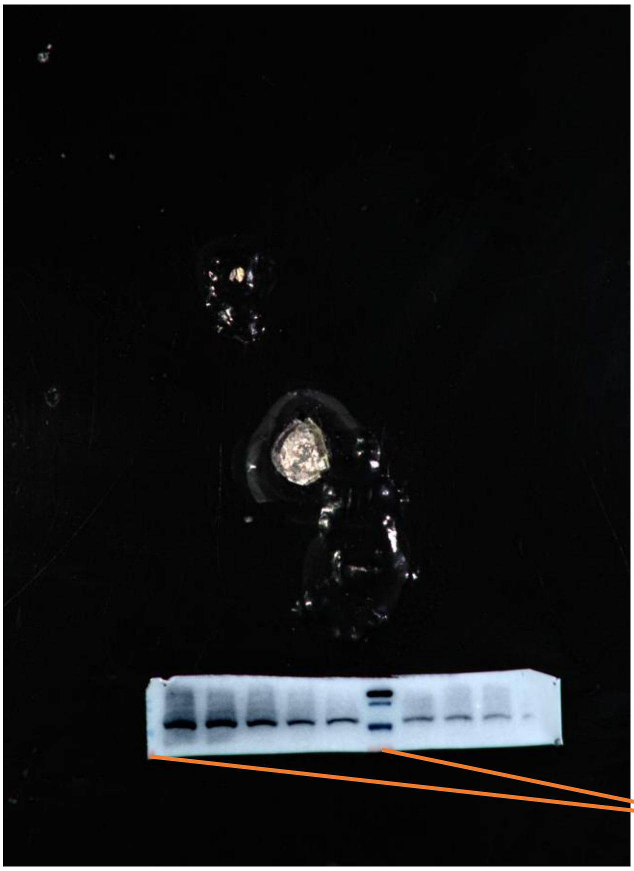
PI3K(110KDa)
Marker(LOT:#26617,red means 70KDa, green means 10KDa)

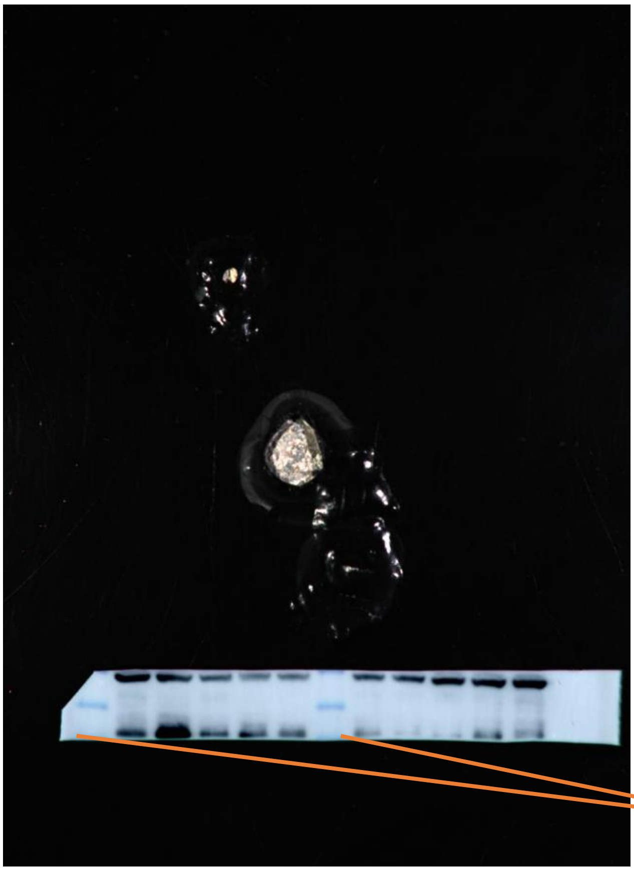

Bax(21KDa)

Marker(LOT:#26617,red means 70KDa, green means 10KDa)

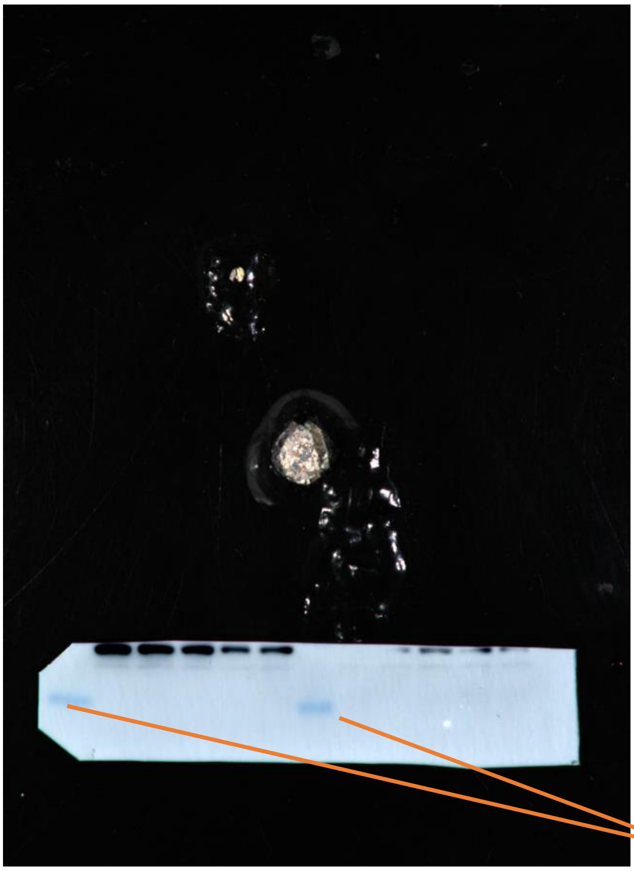

Bcl-2(21KDa)

Marker(LOT:#26617,red means 70KDa, green means 10KDa)

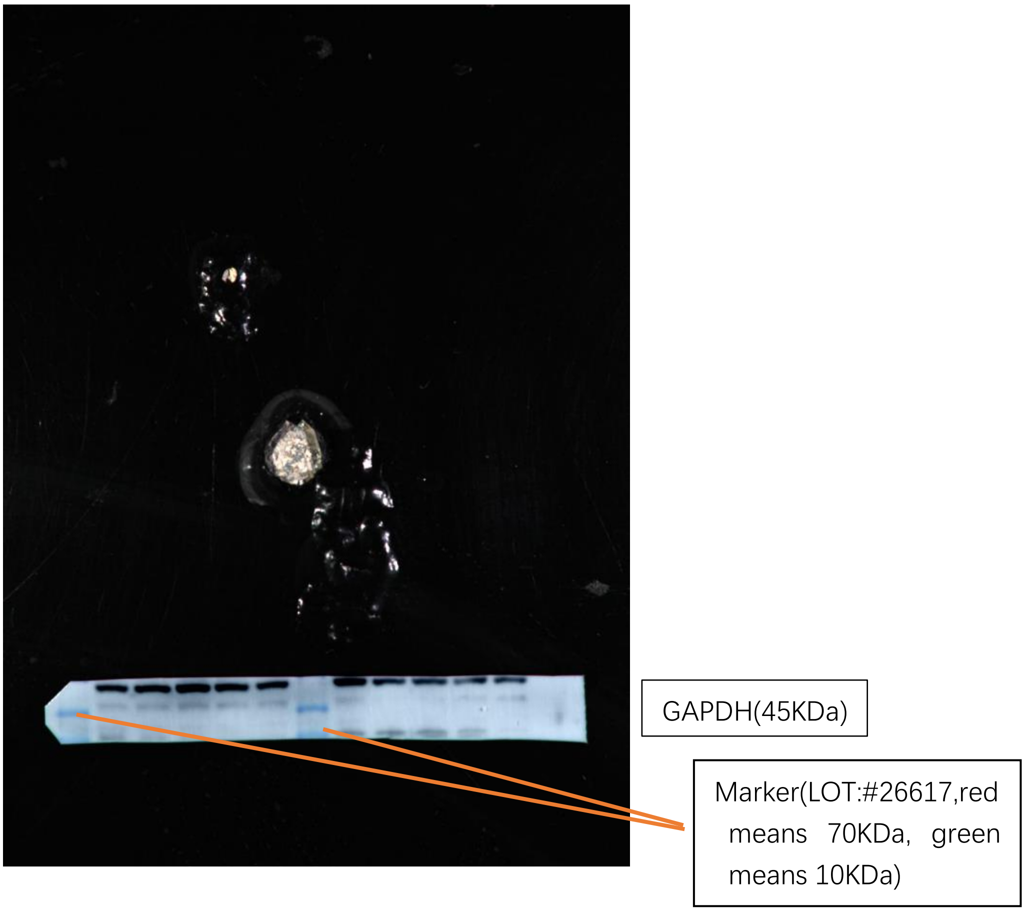
GAPDH(45KDa)
Marker(LOT:#26617,red means 70KDa, green means 10KDa)

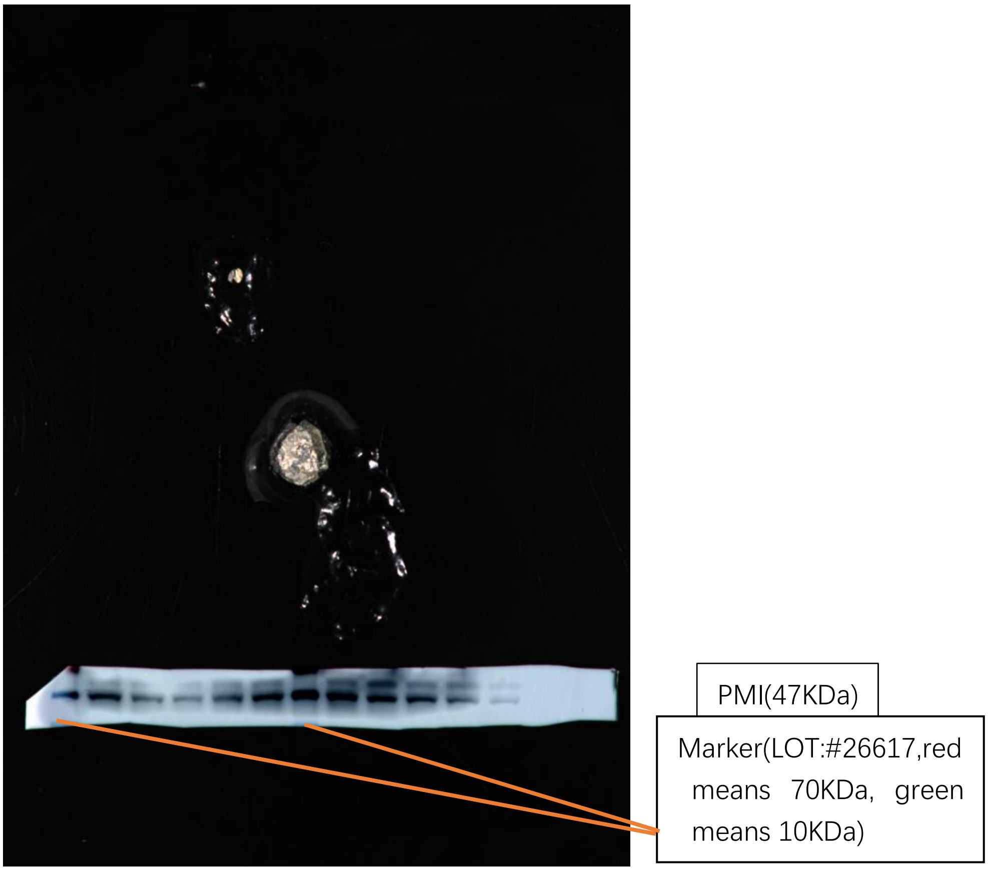
PMI(47KDa)
Marker(LOT:#26617,red means 70KDa, green means 10KDa)

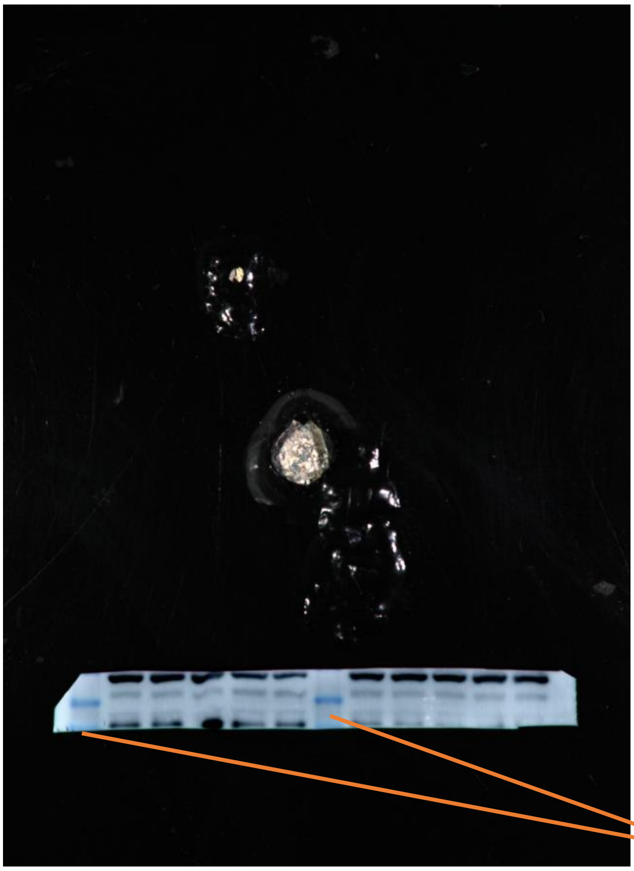

GAPDH(45KDa)

Marker(LOT:#26617,red means 70KDa, green means 10KDa)

2. In vivo

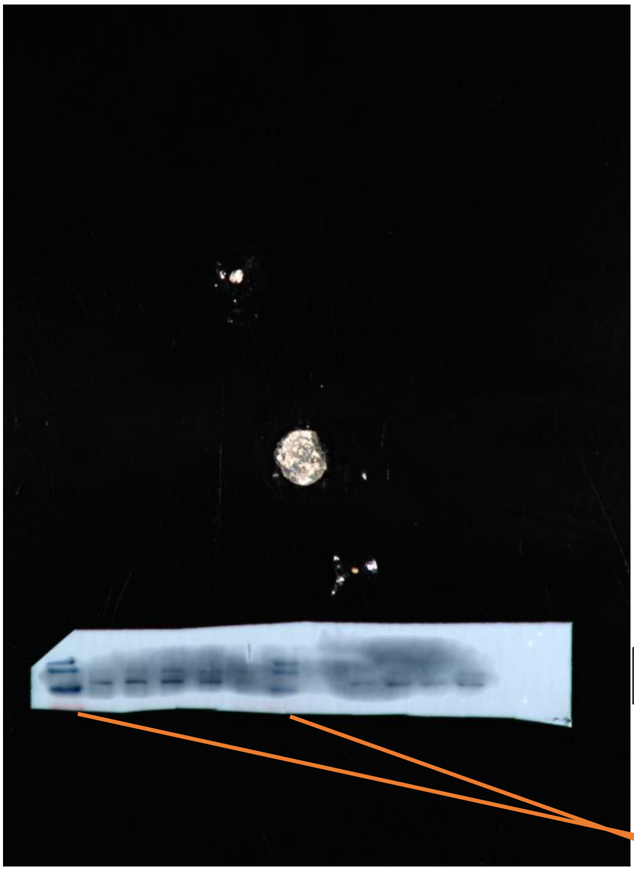

ABCG2(72KDa)

Marker(LOT:#26617,red means 70KDa, green means 10KDa)

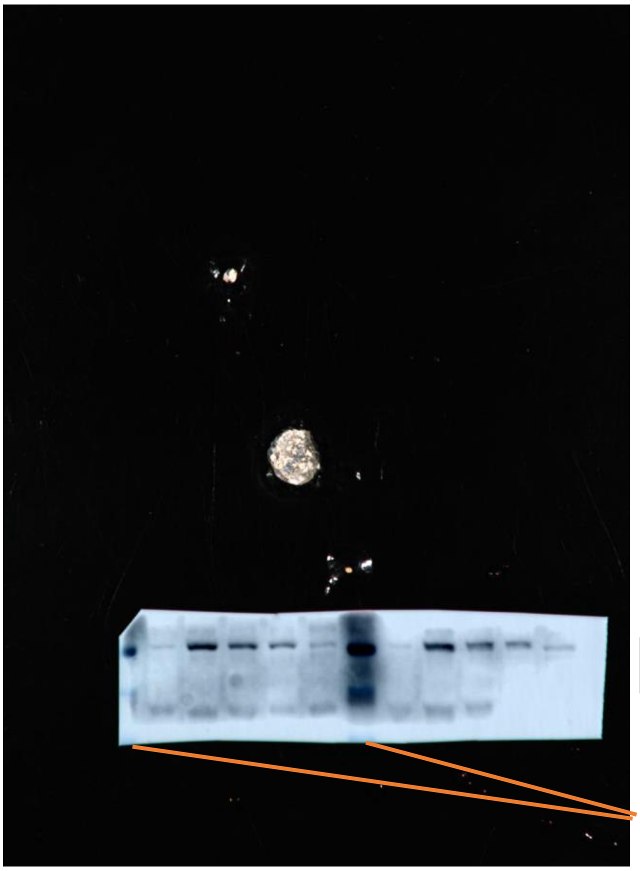

PMI(47KDa)

Marker(LOT:#26617,red means 70KDa, green means 10KDa)

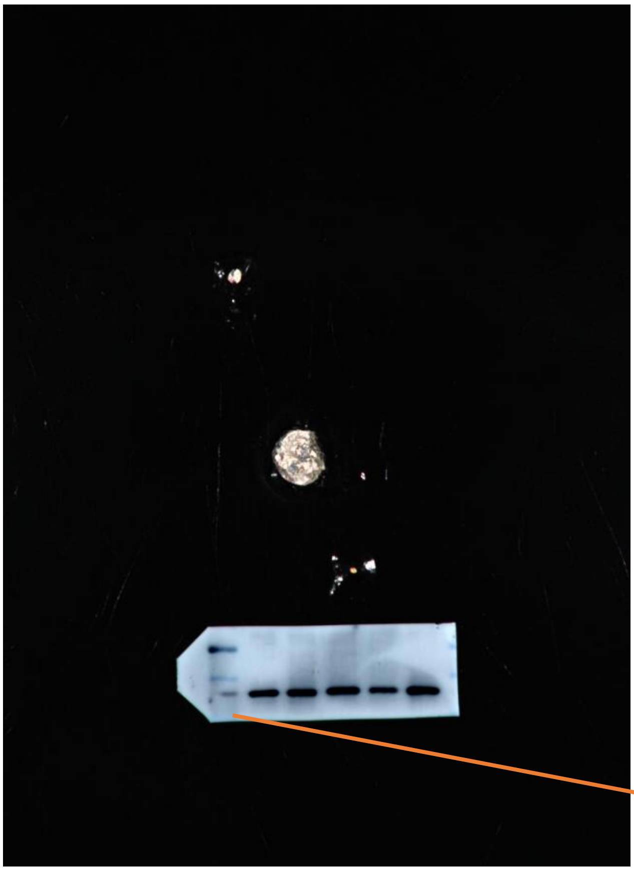

GAPDH(45KDa)

Marker(LOT:#26617,red means 70KDa, green means 10KDa)
